# Correction: Paper-based ELISA diagnosis technology for human brucellosis based on a multiepitope fusion protein

**DOI:** 10.1371/journal.pntd.0011079

**Published:** 2023-01-24

**Authors:** Dehui Yin, Qiongqiong Bai, Xiling Wu, Han Li, Jihong Shao, Mingjun Sun, Hai Jiang, Jinpeng Zhang

The last author’s name is spelled incorrectly. The correct name is: Jinpeng Zhang.
