# Supplementary figures and images for: Paper-based ELISA diagnosis technology for human brucellosis based on a multiepitope fusion protein
Source: PLoS Negl Trop Dis. 2021 Aug 17;15(8):e0009695. doi: 10.1371/journal.pntd.0009695 (PMC8396774; doi:10.1371/journal.pntd.0009695)

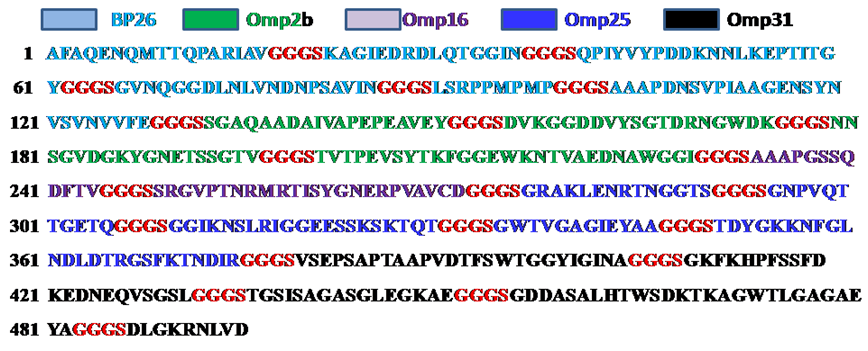

Supplement: S1 Fig — (TIF) [file pntd.0009695.s001.tif]

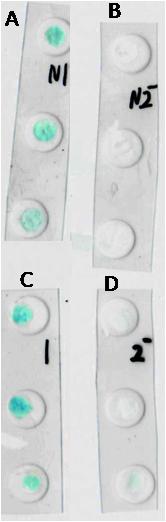

Supplement: S2 Fig — (A) Positive of nano-p-ELISA. (B) Negative of nano-p-ELISA. (C) Positive of tra-p-ELISA. (D) Negative of tra-p-ELISA. (TIF) [file pntd.0009695.s002.tif]
