# Supplementary material for: Paper-based ELISA diagnosis technology for human brucellosis based on a multiepitope fusion protein
Source: PLoS Negl Trop Dis. 2021 Aug 17;15(8):e0009695. doi: 10.1371/journal.pntd.0009695 (PMC8396774; doi:10.1371/journal.pntd.0009695)
Supplement: S1 Table — (DOCX) [file pntd.0009695.s003.docx]

**S1 Table.** Information of the patient

| Order No. | Blood culture serial number | Pathogens |
| --- | --- | --- |
| 1 | 200707B0000035 | Pseudomonas putida |
| 2 | 200706B0000086 | Aeromonas sobria |
| 3 | 200705B0000069 | Staphylococcus haemolyticus |
| 4 | 200706B0000032 | Escherichia coli |
| 5 | 200704B0000100 | Staphylococcus aureus |
| 6 | 200708B0000072 | Klebsiella pneumoniae |
| 7 | 200708B0000168 | Escherichia coli |
| 8 | 200708B0000113 | Staphylococcus saprophyticus |
| 9 | 200708B0000051 | Moraxella osloensis |
| 10 | 200709B0000026 | Staphylococcus hominis |
| 11 | 200709B0000099 | Raoultella ornithinolytica |
| 12 | 200714B0000016 | Escherichia coli |
| 13 | 200713B0000052 | Escherichia coli |
| 14 | 200711b0000011 | Candida parapsilosis |
| 15 | 200715B0000042 | Staphylococcus aureus |
| 16 | 200715B0000014 | Escherichia coli |
| 17 | 200714B0000045 | Staphylococcus epidermidis |
| 18 | 200720B0000036 | Klebsiella pneumoniae |
| 19 | 200721B0000130 | Streptococcus |
| 20 | 200722B0000005 | Pseudomonas aeruginosa |
| 21 | 200722B0000074 | Streptococcus constellatus |
| 22 | 200726B0000056 | Pseudomonas aeruginosa |
| 23 | 200726B0000039 | Rothia mucilaginos |
| 24 | 200726B0000105 | Enterococcus faecium |
| 25 | 200726B0000040 | Staphylococcus hominis |
| 26 | 200725B0000017 | Klebsiella pneumoniae |
| 27 | 200727B0000068 | Escherichia coli |
| 28 | 200727B0000065 | Staphylococcus aureus |
| 29 | 200724B0000116 | Escherichia coli |
| 30 | 200728B0000078 | Enterococcus faecium |
| 31 | 200728B0000030 | Escherichia coli |
| 32 | 200727B0000128 | Escherichia coli |
| 33 | 200801B0000030 | Enterococcus faecium |
| 34 | 200802B0000001 | Staphylococcus aureus |
| 35 | 200801B0000108 | Staphylococcus aureus |
| 36 | 200803B0000007 | Pseudomonas aeruginosa |
| 37 | 200805B0000057 | Staphylococcus aureus |
| 38 | 200804B0000088 | Streptococcus dysgalactiae |
| 39 | 200804B0000051 | Escherichia coli |
| 40 | 200805B0000125 | Escherichia coli |
