# Supplementary material for: Paper-based ELISA diagnosis technology for human brucellosis based on a multiepitope fusion protein
Source: PLoS Negl Trop Dis. 2021 Aug 17;15(8):e0009695. doi: 10.1371/journal.pntd.0009695 (PMC8396774; doi:10.1371/journal.pntd.0009695)
Supplement: S2 Table — (DOC) [file pntd.0009695.s004.doc]

**S2 Table.** The OMPs' Accession Numbers of *Brucella* in NCBI Protein database

| OMPs | Accession Numbers of *Brucella* spp. | | |
| --- | --- | --- | --- |
|  | *B. melitensis* | *B. abortus* | *B. suis* |
| BP26 | AAB38523.1 | AAO39773.1 | KFJ31678.1 |
| Omp16 | AEF59023.1 | AGI97133.1 | AIB29945.1 |
| Omp25 | AEF59022.1 | AFJ79953.1 | AHN46339.1 |
| Omp31 | ACS50328.1 | - | AAL27290.1 |
| Omp2b | AMM72579.1 | SUW28200.1 | SUW48930.1 |
