# Supplementary material for: Paper-based ELISA diagnosis technology for human brucellosis based on a multiepitope fusion protein
Source: PLoS Negl Trop Dis. 2021 Aug 17;15(8):e0009695. doi: 10.1371/journal.pntd.0009695 (PMC8396774; doi:10.1371/journal.pntd.0009695)
Supplement: Attachment — Submitted filename: PNTD-D-21-00330_reviewer-1 30 May 2021.pdf [file pntd.0009695.s010.pdf]

PLOS Neglected Tropical Diseases

Paper-based ELISA diagnosis technology for human brucellosis based on a multiepitope fusion protein

--Manuscript Draft--

**Manuscript Number:**	PNTD-D-21-00330
**Full Title:**	Paper-based ELISA diagnosis technology for human brucellosis based on a multiepitope fusion protein
**Short Title:**	P-ELISA for diagnosing human brucellosis
**Article Type:**	Research Article
**Keywords:**	brucellosis; nano-zinc oxide; p-ELISA; immunodiagnosis
**Abstract:**	Background: At present, as a serious zoonotic infectious disease, the incidence of brucellosis is increasing each year worldwide, exhibiting signs of resurgence. Brucellosis seriously threatens the health of humans, and it is necessary to strengthen the methods utilized for its rapid and accurate diagnosis.<br>Methodology/Principal Findings: Bioinformatic technology was used to predict B-cell epitopes of the main outer membrane proteins of Brucella and subsequently verified the antigenicity of these epitopes. Prepared a Brucella multiepitope fusion protein and verified the antigenicity of the protein by indirect ELISA. Whatman filter paper was then modified with nano-zinc oxide to construct a paper-based ELISA (p-ELISA) technology for the diagnosis of brucellosis. A total of 22 linear B cell epitopes were predicted. Each epitope could recognize some brucellosis sera. The constructed multiepitope fusion protein had good antigenicity and significantly reduced cross-reaction compared with LPS. The sensitivity and specificity of the method were 92.38% and 98.35%, the positive predictive value was 98.26%, and the negative predictive value was 91.67%.<br>Conclusions: A multiepitope fusion protein of Brucella was successfully prepared, and a rapid diagnostic technique for brucellosis was established. This technology has potential application value and can be used for the rapid diagnosis of brucellosis.
**Additional Information:**	
**Question**	**Response**
**Financial Disclosure**<br><br>Enter a financial disclosure statement that describes the sources of funding for the work included in this submission. Review the submission guidelines for detailed requirements. View published research articles from *PLOS NTDs* for specific examples.<br><br>This statement is required for submission and **will appear in the published article** if the submission is accepted. Please make sure it is accurate.	This work was supported by the National Natural Science Foundation of China (Grant number 81802101， received by Dehui Yin). The funders had no role in study design, data collection and analysis, decision to publish, or preparation of the manuscript.

Powered by Editorial Manager® and ProduXion Manager® from Aries Systems Corporation

**Unfunded studies**<br><br>Enter: *The author(s) received no specific funding for this work.*<br><br>**Funded studies**<br><br>Enter a statement with the following details:<br>• Initials of the authors who received each award<br>• Grant numbers awarded to each author<br>• The full name of each funder<br>• URL of each funder website<br>• Did the sponsors or funders play any role in the study design, data collection and analysis, decision to publish, or preparation of the manuscript?<br>• **NO** - Include this sentence at the end of your statement: *The funders had no role in study design, data collection and analysis, decision to publish, or preparation of the manuscript.*<br>• **YES** - Specify the role(s) played.<br><br>* typeset	
**Competing Interests**<br><br>Use the instructions below to enter a competing interest statement for this submission. On behalf of all authors, disclose any competing interests that could be perceived to bias this work—acknowledging all financial support and any other relevant financial or non-financial competing interests.<br><br>This statement **will appear in the published article** if the submission is accepted. Please make sure it is accurate. View published research articles from *PLOS NTDs* for specific examples.	The authors have declared that no competing interests exist.

**NO authors have competing interests**<br><br>Enter: *The authors have declared that no competing interests exist.*<br><br>**Authors with competing interests**<br><br>Enter competing interest details beginning with this statement:<br><br>*I have read the journal's policy and the authors of this manuscript have the following competing interests: [insert competing interests here]*<br><br>* typeset<br><br>This statement is required for submission and **will appear in the published article** if the submission is accepted. Please make sure it is accurate and that any funding sources listed in your Funding Information later in the submission form are also declared in your Financial Disclosure statement.	
**Data Availability**<br><br>Authors are required to make all data underlying the findings described fully available, without restriction, and from the time of publication. PLOS allows rare exceptions to address legal and ethical concerns. See the PLOS Data Policy and FAQ for detailed information.	Yes - all data are fully available without restriction

Powered by Editorial Manager® and ProduXion Manager® from Aries Systems Corporation

A Data Availability Statement describing where the data can be found is required at submission. Your answers to this question constitute the Data Availability Statement and **will be published in the article**, if accepted.<br><br>**Important:** Stating 'data available on request from the author' is not sufficient. If your data are only available upon request, select 'No' for the first question and explain your exceptional situation in the text box.<br><br>Do the authors confirm that all data underlying the findings described in their manuscript are fully available without restriction?	
**Describe where the data may be found in full sentences. If you are copying our sample text, replace any instances of XXX with the appropriate details.**<br><br>• If the data are **held or will be held in a public repository**, include URLs, accession numbers or DOIs. If this information will only be available after acceptance, indicate this by ticking the box below. For example: *All XXX files are available from the XXX database (accession number(s) XXX, XXX.*).<br>• If the data are all contained **within the manuscript and/or Supporting Information files**, enter the following: *All relevant data are within the manuscript and its Supporting Information files.*<br>• If neither of these applies but you are able to provide **details of access elsewhere**, with or without limitations, please do so. For example:<br><br>*Data cannot be shared publicly because*	All relevant data are within the manuscript and its Supporting Information files.

of [XXX]. Data are available from the XXX Institutional Data Access / Ethics Committee (contact via XXX) for researchers who meet the criteria for access to confidential data.<br><br>*The data underlying the results presented in the study are available from (include the name of the third party and contact information or URL).*<br>• This text is appropriate if the data are owned by a third party and authors do not have permission to share the data.<br><br>* typeset	
Additional data availability information:	

Powered by Editorial Manager® and ProduXion Manager® from Aries Systems Corporation

Manuscript

Click here to access/download;Manuscript;edited manuscript0301.docx

Paper-based ELISA diagnosis technology for human brucellosis based on a multiepitope fusion protein

Running head：P-ELISA for diagnosing human brucellosis

Dehui Yin[1], Qiongqiong Bai[1], Xiling Wu[1], Han Li[2], Jihong Shao[1], Mingjun Sun[3#*], Hai Jiang[4#*], Jingpeng Zhang[1#*]

[1] Key Lab of Environment and Health, School of Public Health, Xuzhou Medical University, Xuzhou, 221004, China

[2]Department of Infection Control, the First Hospital of Jilin University, Changchun, 130021, China

[3] Laboratory of Zoonoses, China Animal Health And Epidemiology Center, Qingdao, 266032, China

[4]State Key Laboratory for Infectious Disease Prevention and Control, Collaborative Innovation Center for Diagnosis and Treatment of Infectious Diseases, National Institute for Communicable Disease Control and Prevention, Chinese Center for Disease Control and Prevention, Beijing, China

# These authors contributed equally to this work

*Correspondence:

Laboratory of Zoonoses, China Animal Health And Epidemiology Center, No.369 Nanjing Road, Qingdao, 266032, China. E-mail: sunmingjun@cahec.cn

State Key Laboratory for Infectious Disease Prevention and Control, Chinese Center for Disease Control and Prevention, No. 155 Changbai Road, Beijing, 102206, China.

E-mail: jianghai@icdc.cn.
School of Public Health, Xuzhou Medical University, No. 129 Tongshan Road, Xuzhou, 221004, China. E-mail: xiaopangpeng@126.com

Abstract

Background: At present, as a serious zoonotic infectious disease, the incidence of brucellosis is increasing each year worldwide, exhibiting signs of resurgence. Brucellosis seriously threatens the health of humans, and it is necessary to strengthen the methods utilized for its rapid and accurate diagnosis.

Methodology/Principal Findings: Bioinformatic technology was used to predict B-cell epitopes of the main outer membrane proteins of Brucella and subsequently verified the antigenicity of these epitopes. Prepared a Brucella multiepitope fusion protein and verified the antigenicity of the protein by indirect ELISA. Whatman filter paper was then modified with nano-zinc oxide to construct a paper-based ELISA (p-ELISA) technology for the diagnosis of brucellosis. A total of 22 linear B cell epitopes were predicted. Each epitope could recognize some brucellosis sera. The constructed multiepitope fusion protein had good antigenicity and significantly reduced cross-reaction compared with LPS. The sensitivity and specificity of the method were 92.38% and 98.35%, the positive predictive value was 98.26%, and the negative predictive value was 91.67%.

Conclusions: A multiepitope fusion protein of Brucella was successfully prepared, and a rapid diagnostic technique for brucellosis was established. This technology has potential application value and can be used for the rapid diagnosis of brucellosis.

Keywords: brucellosis; nano-zinc oxide; p-ELISA; immunodiagnosis

Author Summary

Brucellosis has caused tremendous economic losses in agriculture and husbandry

in various countries. Therefore, developing rapid, sensitive and specific diagnostic techniques for brucellosis has become critical brucellosis research. In this study, a low-cost diagnostic technique for use in resource-constrained settings was established. We used immunoinformatic technology to predict the B cell epitopes in the major outer membrane proteins of Brucella, synthesized polypeptides and coupled them with KLH, screened these polypeptides by iELISA methods, selected effective polypeptides as diagnostic antigens, and established a p-ELISA for brucellosis diagnosis based on a multiepitope fusion protein that can be used to assess the serum of human.

Introduction

Brucellosis is a reemerging zoonotic infectious disease. In recent years, the incidence rate has been increasing yearly. It not only seriously threatens the health of the people but also causes huge economic losses to the animal husbandry industry. Brucellosis has a complex condition and a long course of disease, causing a huge economic burden and a waste of medical resources in countries all over the world, especially after misdiagnosis, which will increase the treatment cost[1]. Therefore, the rapid and accurate diagnosis of brucellosis is necessary. At present, the diagnostic methods for brucellosis include traditional pathogenic detection, serological diagnostic techniques and molecular biology methods[2,3].

The traditional isolation culture method requires complicated laboratory and medium conditions, which need to be carried out in a P3 laboratory, and the culture time is long, usually several weeks[4]. Molecular biology methods such as PCR technology have specific and rapid characteristics, but nucleic acid contamination will cause false positive results, the sensitivity and specificity are not high, and it requires relatively expensive instruments and professional operation, which is not conducive to popularization in grassroots units[5]. Serological diagnostic techniques mainly include the agglutination test, complement fixation test (CFT), enzyme-linked immunosorbent assay (ELISA), immunochromatographic diagnostic test (ICDT), and fluorescence polarization assay (FPA), which are currently the commonly used screening methods for brucellosis[6]. This method has the advantages of high sensitivity and short operation time, but the diagnosis of serological methods is

greatly affected by antigens. The key technology to improve the sensitivity and specificity of serological diagnostic methods is to find suitable diagnostic antigens. Paper-based enzyme-linked immunosorbent assay (p-ELISA) is an emerging detection technology. It has the same principle as the traditional 96-well plate ELISA except that it uses paper as a solid phase carrier. Due to its small reagent dosage, rapid detection, low cost, and lack of need for special equipment, it has attracted increasing clinical attention[7,8]. The main advantages of paper as a carrier are as follows: wide range of paper sources and low cost; through capillary action, no external force is needed to make the liquid flow; good biocompatibility; small reagent loading volume; and easy to use and carry[9].

In this study, we used immunoinformatic technology to predict the B cell epitopes in the major outer membrane proteins of Brucella, synthesized polypeptides and coupled them with KLH, screened these polypeptides by traditional ELISA methods, selected effective polypeptides as diagnostic antigens, and established a nano-ZnO-modified p-ELISA for brucellosis diagnosis based on a multiepitope fusion protein.

Methods

Human serum samples

There were 121 human brucellosis sera (gifted by the School of Public Health of Jilin University), 90 control sera, including 50 healthy sera and 40 patient sera (confirmed by blood culture to be infected with other pathogens, collected by the infection department of the First Clinical Hospital of Jilin University; information on

the patients is shown in Table S1). All experiments involving human or animal samples were fully compliant with ethical approval granted by the Animal Care and Ethics Committee of Xuzhou Medical University.

Selection of the outer membrane proteins of Brucella

The antigenicity of the outer membrane proteins of Brucella was investigated by consulting the literature (https://www.ncbi.nlm.nih.gov/protein/). The amino acid sequence was obtained, and the conservation of the amino acid sequence was analyzed by BLAST.

B cell epitope prediction and peptide synthesis

The conserved amino acid sequence of the Brucella outer membrane protein was used to predict B cell epitopes by using the B cell epitope prediction tool bepipred linear epitope prediction 2.0 in IEDB (http://tools.iedb.org/bcell/). The predicted B cell epitopes were delivered to SGS and coupled with keyhole limpet hemocyanin (KLH). The predicted B cell epitope was handed over to Sangon Biotech (Shanghai, China) for synthesis and coupled with keyhole limpet hemocyanin (KLH).

Screening of peptide epitopes

To screen effective peptide epitopes, we verified the antigenicity of predicted epitopes by indirect ELISA. The experimental procedure was as follows: peptides coupled to KLH were diluted with carbonate buffer (pH = 9.6) to final concentrations of 30 μg/mL and 100 μL/well in a 96-well plate (Corning, USA) and incubated overnight at 4°C. Next, 300 μL/well of blocking solution (PBS containing 5% skimmed milk powder) was incubated at 37°C for 1 h, and the cells were washed 3

times with PBST (PBS containing 0.05% Tween 20). Afterwards, 100 μL/well of 1:400 serum was added and incubated at 37℃ for 1 h followed by washing 3 times with PBST. Next, 1:5000 diluted HRP-labeled protein G (Thermo, USA) was added; it was reacted at room temperature for 15 min and washed again with PBST 3 times. Next, 100 μL of TMB substrate solution was added to each well and reacted for 15 min at room temperature followed by the addition of 50 μL of stop solution (2 M H_2SO_4). The optical density was measured at 450 nm (OD450) using an ELISA plate reader (BioTek, USA). At the same time, KLH (30 μg/mL) and lipopolysaccharide (LPS, 1 μg/mL, provided by China Animal Health and Epidemiology Center (Qingdao, China)) were used as blank carriers and positive antigen controls to detect serum.

Fusion protein preparation

The selected effective peptides are connected in series, and the adjacent two peptide chains are connected with the linker 'GGGS'. The plasmid was constructed by full gene synthesis, subcloned into the expression vector pET-21a (Sangon Biotech, Shanghai, China) and further transformed into E. coli competent BL21(DE3) cells (Sangon Biotech, Shanghai, China). The cells were cultured, IPTG was used to induce expression, bacteria were collected, the protein was purified, and the target protein was verified by SDS-PAGE and Western blotting. The specific steps are described below.

After transferring the recombinant plasmid into BL21(DE3), 800 μL of nonresistant LB medium was added, followed by incubation at 37°C for 45 min and

centrifugation at 5000 rpm for 3 min. Most of the supernatant was discarded (leave approximately 100-150 μL), the bacteria were resuspended, the LB plate with corresponding resistance was selected, and it was coated. After air-drying, it was inverted and cultured overnight in a 37°C incubator. The monoclonal colonies on the plate were chosen, placed into 10 mL of LB liquid medium and incubated at 37°C and 200 rpm. The cultured bacterial solution was transferred to 750 mL of LB liquid medium at 37°C and 200 rpm, cultured to OD_{600}=0.6-0.8 with IPTG (0.5 mM) at 16°C and induced overnight. Then, the cells were centrifuged at 6000 rpm for 5 min, the supernatant was discarded, and the bacteria were collected. Bacteria were blown away with 20-30 mL 10 mM Tris-HCl (pH = 8.0) solution and ultrasonically broken (500 W, 60 times, 10 s each time, 15 s interval). After sonication, 100 μL of the bacterial suspension was centrifuged at 12000 rpm for 10 min, and 50 μL of supernatant was transferred to another EP tube. After the supernatant was removed, the precipitate was blown away with 50 μL of 10 mM Tris-HCl (pH = 8.0) solution. SDS-PAGE and Western blotting were used to detect protein expression. A nickel column (Ni Sepharose 6 Fast Flow, GE Healthcare) for affinity chromatography was used for protein purification. Taking 5 mL of Ni-NTA, the equilibrium column was washed with 5 times the column bed volume of binding buffer at a flow rate of 5 mL/min. The crude protein was incubated with the equilibrated column packing for 1 h; the incubated product was loaded onto the column and the effluent liquid was collected; the equilibrium column was washed with binding buffer; the column was washed with washing buffer, and the effluent liquid was collected; with the column

was eluted with elution buffer, and the effluent liquid was collected; and the crude protein was treated, washed with effluent and eluted with effluent separately, followed by sample preparation and, SDS-PAGE and WB detection. The concentrated protein was divided into 1 mL/tube and stored at -80°C.

Antigenicity identification of fusion protein

Indirect ELISA was used to verify the antigenicity of the fusion protein. The procedure was as follows: 96-well microtiter plates were coated with 1 μg/well fusion protein at 4°C overnight, and then, 5% skimmed milk powder was blocked at 37°C for 2 h. Serum was diluted 1:400 and added at 100 μL per well, followed by incubation at 37°C for 1 h. HRP-labeled protein G was diluted with 1:8000, added at 100 μL/well, incubated at 37°C for 1 h, and finally developed with TMB substrate solution for 15 min. For termination, 2 M H_2SO_4 was used, and the OD450 was measured. After each step, the cells were washed with PBST (0.05% Tween-20 in PBS) 3 times.

Synthesis of nano-ZnO and paper modification

ZnO nanorods were synthesized on Whatman No. 1 filter paper by a hydrothermal method[10]. The steps were as follows: Whatman filter paper was soaked in 100 mm zinc acetate solution for 60 s and then annealed at 100 ℃ for 1 h to form a seed layer (seed layer). Then, the filter paper with a seed layer was transferred to a hydrothermal reaction vessel containing an equimolar solution (100 mm, pH = 6.5) of hexamethylenetetramine (HMTA, Sigma) and zinc nitrate ($Zn(NO_3)_2.6H_2O$, Sigma). ZnO nanorods were synthesized at 90 ℃ for 5 h. Next, Whatman filter paper

with ZnO nanorods was immersed in anhydrous toluene solution (Sigma) with 1% APTES (Sigma) for 5 min, heated and dried at 100 ℃ for 15 min, and silanized. Scanning electron microscopy (SEM, JSM-7500F), X-ray diffraction (XRD, Bruker D8) and X-ray photoelectron spectroscopy (XPS, Escalable250Xi) were used to characterize the structure and surface of the paper. Whatman filter paper modified with ZnO nanorods was punched into circular paper pieces with a diameter of 10 mm by a punch, and A4 plastic packaging paper was punched into small holes with a diameter of 6 mm by a punch. The 10 mm filter paper pieces were placed in the center of the 6 mm holes of the plastic packaging paper, fixed by a plastic packaging machine, and cut into small strips with 3 holes for standby.

Establishment of p-ELISA

Five microliters of fusion protein solution was placed in each well (30 μg/mL in PBS), incubated at room temperature for 30 min, washed with 20 μL of deionized water 3 times, and blocked with 20 μL of 5% skimmed milk powder at room temperature for 15 min; PBST was used for washing 3 times, and 5 μL of serum was added (diluted with 1:400); PBST was used for washing 3 times, and 5 μL of HRP labeled protein G was added (diluted with 1:8000), followed by incubation at room temperature for 210 s; and PBST was used for washing 3 times, 5 μL of TMB substrate solution was developed for 10 min, and an HP Laser Jet Pro MFP M227 was used for scanning to obtain the image. ImageJ software carries out gray intensity analysis for quantitative analysis.

Traditional p-ELISA

To compare with the nanomodified p-ELISA (nano-p-ELISA) method, we also performed the traditional p-ELISA (tra-p-ELISA) method. The specific steps have been described in the literature[11]. Five microliters of chitosan was added to deionized water (0.25 mg/mL) and placed onto Whatman No.1 filter paper followed by drying at room temperature; then 5 μL of 2.5% glutaraldehyde solution (PBS) was added, followed by resting at room temperature for 2 h and washing with 20 μL of deionized water twice. The remaining steps are same as described in section 2.8.

Statistical analysis

Dot plot and receiver operating characteristic (ROC) curve analyses were performed using GraphPad Prism version 6.05 for Windows. The OD450 and gray intensity were determined by Student's t-test (unpaired t-test). P-values < 0.05 were considered to indicate significant differences.

Results

Brucella outer membrane protein epitope prediction and peptide synthesis

Five highly conserved proteins, omp16, omp25, omp31, omp2b and BP26, were selected. All information on Brucella species and protein accession numbers are shown in Table S2. A total of 22 epitopes were predicted for the selected proteins, and the detailed epitope information is shown in Table 1. The 22 polypeptide epitopes were synthesized and coupled to KLH. KLH was uniformly coupled to the tail end of the polypeptide (right side). Each polypeptide (10 mg) was coupled to 10 mg of KLH, and the purity was >90%.

Table 1 Detailed information of 22 predicted B cell epitopes

Protein	Epitope (amino acid sequence)	Start-end position	Peptide ID
BP26	AFAQENQMTTQPARIAV	26-42	P19266-1
	KAGIEDRDLQTGGIN	88-100	P19266-2
	QPIYVYPDDKNNLKEPTITGY	104-124	P19266-3
	GVNQGGDLNLVNDNPSAVIN	151-170	P19266-4
	LSRPPMPMP	204-212	P19266-5
	AAAPDNSVPIAAGENSYNVSVNVVFE	223-248	P19266-6
Omp2b	SGAQAADAIVAPEPEAVEY	31-49	P19266-7
	DVKGGDDVYSGTDRNGWDK	79-97	P19266-8
	NNSGVDGKYGNETSSGTV	129-146	P19266-9
	TVTPEVSYTKFGGEWKNTVAEDNAWGGI	341-368	P19266-10
Omp16	AAAPGSSQDFTV	44-55	P19266-11
	SRGVPTNRMRTISYGNERPVAVCD	125-148	P19266-12
Omp25	GRAKLENRTNGGTS	56-69	P19266-13
	GNPVQTTGETQ	115-125	P19266-14
	GGIKNSLRIGGEESSKSKTQT	154-174	P19266-15
	GWTVGAGIEYAA	175-186	P19266-16
	TDYGKKNFGLNDLDTRGSFKTNDIR	199-223	P19266-17
Omp31	VSEPSAPTAAPVDTFSWTGGYIGINA	24-49	P19266-18
	GKFKHPFSSFDKEDNEQVSGSL	53-75	P19266-19
	TGSISAGASGLEGKAE	112-127	P19266-20

GDDASALHTWSDKTKAGWTLGAGAEYA	168-194	P19266-21
DLGKRNLVD	109-217	P19266-22

Peptide screening

The KLH-conjugated polypeptide was used as the antigen, and the indirect ELISA method was used to verify the antigenicity of the polypeptide. Positive serum was used to screen the polypeptide. The results of iELISA show that each peptide can recognize some sera, and the recognition ability is quite different (see Fig 1).

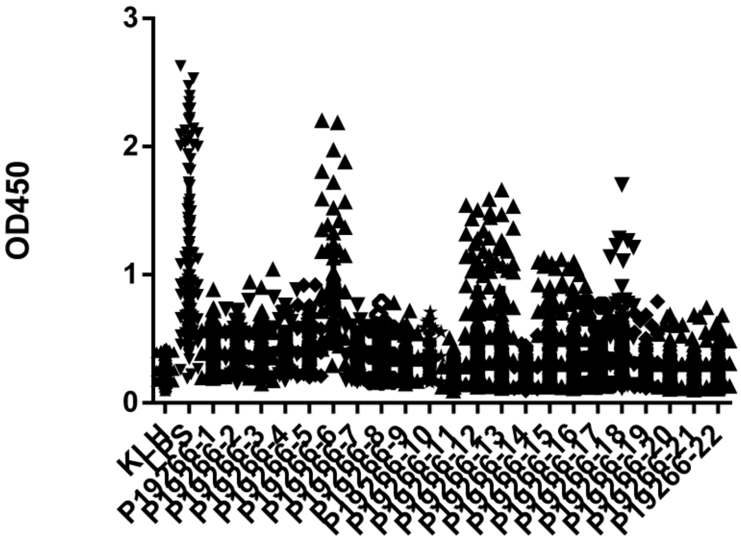

Fig 1. The results of iELISA of each peptide identification-positive brucellosis serum

Fusion protein preparation

SDS-PAGE identification results showed that after 0.5 mM IPTG-induced expression overnight, the expression of the fusion protein reached an ideal amount, and the expression molecular weight was approximately 66 kDa. The results are shown in Fig. 2A and Fig. 2B. After Western blot identification and analysis, the

molecular weight of the histidine tag was consistent with the SDS-PAGE results, and after mass spectrometry verification, it was confirmed that the expressed protein was the target protein. The detailed results are shown in Fig. 2C.

Antigenicity identification of fusion protein

The antigenicity of the purified fusion protein was verified by iELISA. Compared with the LPS antigen, the area under the diagnostic curve of the fusion protein was 0.9877 (95% CI: 0.9758 to 0.9996), while the area under the LPS curve was 0.9174 (95% CI: 0.8796 to 0.9552) (the results are shown in Fig. 3). The optimal cutoff value was calculated by the Youden index, the positive predictive value and negative predictive value of the fusion protein were higher than those of LPS, and the diagnostic predictive value of the two antigens under this cutoff value was analyzed (Table 2).

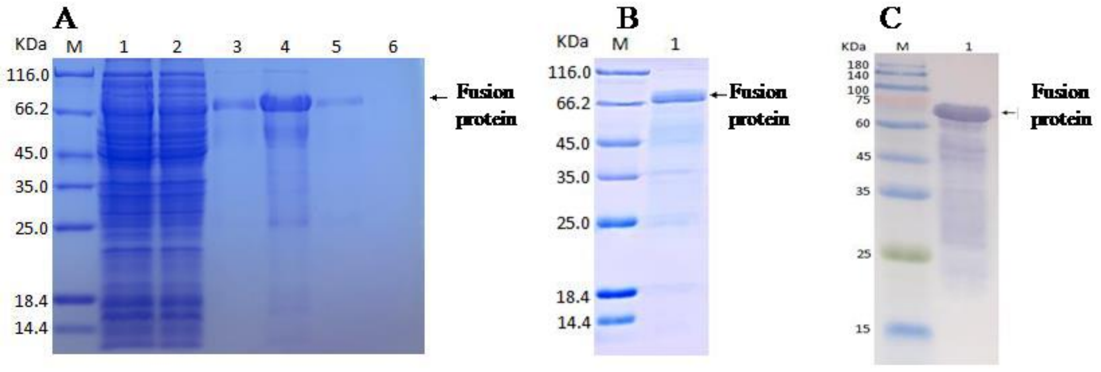

Fig. 2 SDS-PAGE and Western blot analysis of fusion proteins. (A) SDS-PAGE identification results after IPTG-induced expression overnight (M, marker; lane1, loading solution; lane2, flow-through solution; lane3-4, 20 mM imidazole elution fraction; lane5, 50 mM imidazole elution fraction; lane6, 500 mM imidazole elution

fraction). (B) SDS-PAGE identification results of purified fusion protein (M, marker; lane1, purified protein). (C) Western blot results of purified protein (M, marker; lane1, purified protein).

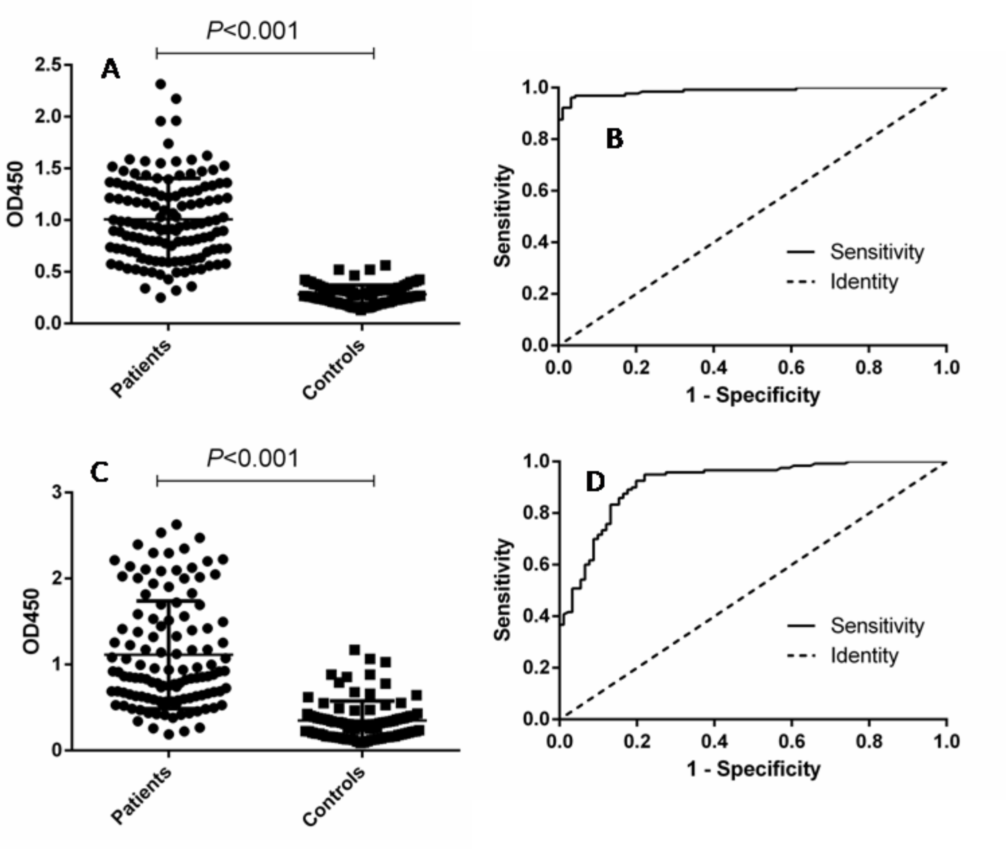

Fig. 3 ELISA analysis of human serum samples. (A**)** Dotplot of the fusion protein ELISA assay. (B) ROC analysis of fusion protein IELISA assay results. (**C)** Dotplot of the LPS antigen ELISA assay. (D) ROC analysis of LPS antigen ELISA assay results.

Table 2. Positive and negative predictive values of the test calculated for different cutoff values

Cutoff value	Positive		Negative		PPV (%)	NPV (%)
	TP	FN	TN	FP		

≥0.470 (fusion protein)	117	4	87	3	95.90	95.51
≥0.4095 (LPS)	115	6	70	20	85.19	92.10
≥50.98 (nano-p-ELISA)	113	8	88	2	98.26	91.67
≥45.66 (tra-p-ELISA)	113	8	87	3	97.41	91.58

TP, true positives; TN, true negatives; FP, false positives; FN, false negatives; PPV, positive predictive value (TP/TP+FP)×100; NPV, negative predictive value (TN/TN+FN) ×100.

Synthesis and characterization of nano-ZnO

Scanning electron microscopy showed that we synthesized nano-zinc oxide on the surface of Whatman filter paper (Fig. 4B). At the same time, XRD results showed that the functionalization of nano-zinc oxide was successfully performed (Fig. 4A). XPS shows that the concentration of Zn atoms is 40.79% and the concentration of oxygen atoms is 59.21%, which further indicates that the concentration of zinc and oxygen in the sample is ZnO.

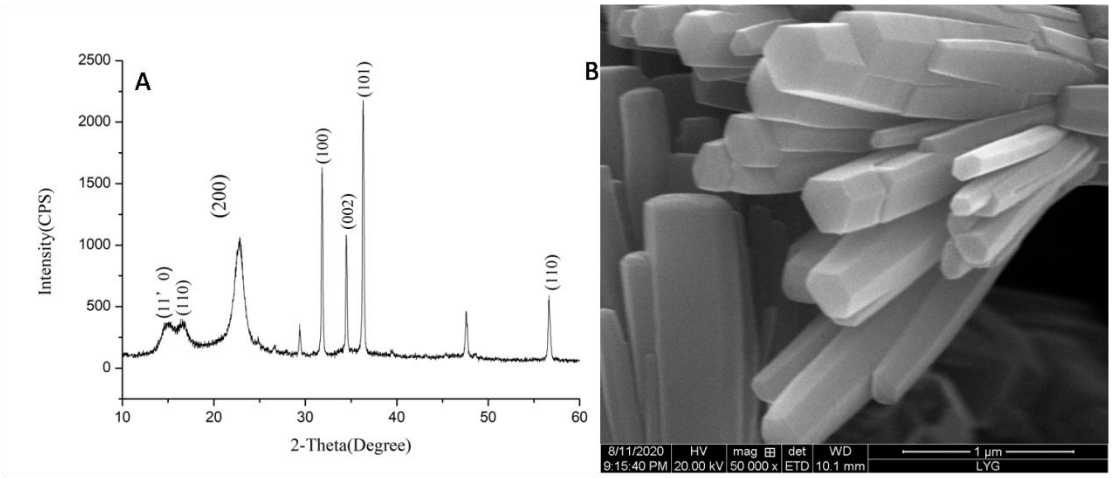

Fig. 4 Nano-ZnO characterization results. (A) XRD results. (B) Scanning electron microscope results.

Evaluation of the diagnostic effect of p-ELISA

A total of 211 sera were detected by the p-ELISA method modified by nano-ZnO (nano-p-ELISA) and the p-ELISA method modified by chitosan and glutaraldehyde (tra-p-ELISA) to evaluate the diagnostic effects of the two methods. The gray intensity of 121 brucellosis-positive sera and 90 brucellosis-negative sera was analyzed by ROC curve analysis, and the results of the nano-p-ELISA showed that the area under the curve was 0.9900 (95% CI, 0.9816 to 0.9984), indicating that the diagnosis of this method has a very high accuracy. The optimal cutoff value was 50.98. Under this cutoff value, the sensitivity of this method was 92.38% (95% CI, 0.8554 to 0.9665) and the specificity was 98.35% (95% CI, 0.9416 to 0.9980) (Fig. 5A). Using this optimal cutoff value to further analyze the diagnostic effect, 113 of the 121 positive samples were accurately diagnosed, and 88 of the 90 negative samples were correct. The positive predictive value of the nano-p-ELISA was 98.26%, and the negative predictive value was 91.67% (Table 2). The gray intensities of the positive and negative samples were significantly different ($P<0.001$) (Fig. 5B).

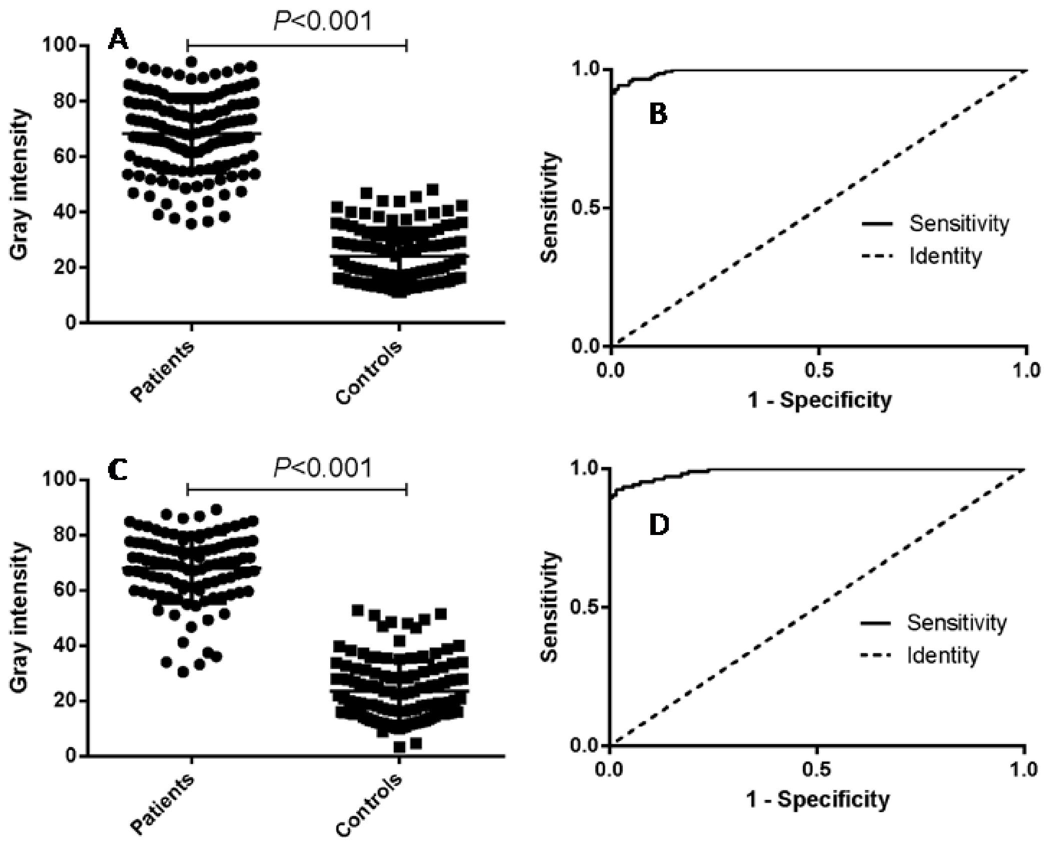

Fig. 5 P-ELISA analysis of human serum samples. (A**)** Dotplot of the nano-p-ELISA assay. (B) ROC analysis of nano-p-ELISA assay results. (**C)** Dotplot of the tra-p-ELISA assay. (D) ROC analysis of tra-p-ELISA assay results.

The results of the tra-p-ELISA showed that the area under the curve was 0.98.94 (95% CI, 0.9817 to 0.9970), indicating that the diagnosis of this method also has high accuracy. The optimal cutoff value is 45.66. Under this cutoff value, the diagnostic sensitivity of this method was 94.24% (95% CI, 0.8897 to 0.9748), and the specificity was 98.26% (95% CI, 0.9386 to 0.9979) (Fig. 5C). With this optimal cutoff value, 113 of the 121 positive samples were accurately diagnosed, and 87 of the 90 negative samples were correctly judged as negative. The positive predictive value of the tra-p-ELISA was 97.41%, and the negative predictive value was 91.58% (Table 2).

The gray intensities of the positive and negative samples were significantly different (P<0.001) (Fig. 5D).

Discussion

The existing diagnostic methods for brucellosis have disadvantages such as complicated operation, long time consumption, low sensitivity, and proneness to cross-reaction[12]. Therefore, a simple, fast and sensitive diagnostic method is sought. Early diagnosis and early treatment of the disease are of great significance to reduce economic loss and medical burden[13]. The development of new diagnostic techniques for brucellosis is of great significance for the prevention and control of brucellosis.

At present, many vaccine studies have shown that many outer membrane proteins in Brucella have strong antigenicity[14-16]. Animal experiments have also shown that these ingredients have a certain immunoprotective effect on Brucella and are good vaccine candidates[17,18]. Therefore, these components also provide a direction for researchers to develop new diagnostic antigens for brucellosis, and the development of immunoinformatic technology provides tools for the development of new diagnostic antigens. Immunoinformatic is based on bioinformatic tools, an emerging science that integrates life sciences, computer science, and mathematics[19,20]. Immunoinformatic technology uses bioinformatic tools to treat pathogens without cultivating them. Processing and analysis of, for instance, the genome and proteome can be used to complete the gene prediction and the prediction of cell epitopes in the protein[21]. Immunoinformatic technology has the advantages of speed and economy

and has been widely used in vaccine design, disease prevention, diagnosis and treatment. In this study, we selected five Brucella antigen proteins, omp16, omp25, omp31, omp2b and BP26, and predicted 22 epitopes. The results of iELISA confirmed that each peptide can recognize a portion of brucellosis sera, but the recognition ability of each polypeptide is limited. Therefore, we concatenated 22 epitopes to synthesize a fusion protein. Using the fusion protein as an antigen, the ability to recognize serum is greatly improved. Compared with LPS, the use of fusion proteins can significantly reduce serological cross-reactions.

P-ELISA has attracted the attention of many researchers due to its advantages, such as strong specificity, simplicity, rapidity, portability, and low cost, especially in the fields of medical testing, environmental testing and food safety analysis[22,23]. It combines the advantages of traditional ELISA and paper and provides a new method and new idea for resource-poor areas and point-of-care testing. At present, the commonly used p-ELISA is a method of modifying the surface of paper with glutaraldehyde and chitosan. Currently, nanomaterials have the characteristics of a large specific surface area and quantum size effect and have been widely used in the fields of biology and medicine[24,25]. The modification of nanomaterials on the surface of paper can increase the surface area of the paper and establish a p-ELISA diagnostic method for nanomaterial modification[10,26]. In this study, we modified the surface of paper with nano-ZnO and used a synthetic fusion protein as the antigen to establish a new diagnostic technique for brucellosis. The diagnostic effect is good, and it can be modified with glutaraldehyde and chitosan. The p-ELISA diagnostic

method is comparable. In addition, p-ELISA paper modified by nanomaterials can be stored at room temperature for a long time, and we also found in experiments that the color development time of p-ELISA modified by nanomaterials can be significantly extended, which may be due to the antioxidant effect of nanomaterials, thus extending the effective time of the color reagent. Compared with the traditional ELISA method, the p-ELISA diagnostic method uses very few reagents and shortens the time for the entire process. It is a relatively promising on-site rapid detection technology.

In summary, using bioinformatic technology combined with nanomaterials, this performance has established a new type of brucellosis diagnostic technology, which has good potential application value. However, the brucellosis sera selected in this study were all clinically screened positive sera, and the number was limited. The diagnostic validity of this method requires a large number of clinical random samples for verification.

Acknowledgments

Not applicable

Competing interests

The authors declare that they have no competing interests

Funding

This work was supported by the National Natural Science Foundation of China (Grant number 81802101). The funders had no role in study design, data collection and analysis, decision to publish, or preparation of the manuscript.

References

1. Olsen SC, Palmer MV. Advancement of knowledge of Brucella over the past 50 years. Vet Pathol. **2014**; 51:1076-89.

2. Pappas G, Papadimitriou P, Akritidis N, Christou L, Tsianos EV. The new global map of human brucellosis. Lancet Infect Dis. **2006**;6:91-9.

3. Dean AS, Crump L, Greter H, Schelling E, Zinsstag J. Global burden of human brucellosis: a systematic review of disease frequency. PLoS Negl Trop Dis. **2012**;6:e1865.

4. Araj GF. Update on laboratory diagnosis of human brucellosis. Int J Antimicrob Agents. **2010**;36 Suppl 1:12-7.

5. Zeybek H, Acikgoz ZC, Dal T, Durmaz R. Optimization and validation of a real-time polymerase chain reaction protocol for the diagnosis of human brucellosis. Folia Microbiol (Praha). **2020**;65:353-61.

6. Yagupsky P, Morata P, Colmenero JD. Laboratory Diagnosis of Human Brucellosis. Clin Microbiol Rev. **2019**;33:e00073-19.

7. Zhao Y, Zeng D, Yan C, Chen W, Ren J, Jiang Y, et al. Rapid and accurate detection of Escherichia coli O157:H7 in beef using microfluidic wax-printed paper-based ELISA. Analyst. **2020**;145:3106-15.

8. Kasetsirikul S, Umer M, Soda N, Sreejith KR, Shiddiky MJA, Nguyen NT. Detection of the SARS-CoV-2 humanized antibody with paper-based ELISA. Analyst. **2020**;145:7680-86.

9. Cheng CM, Martinez AW, Gong J, Mace CR, Phillips ST, Carrilho E, et al. Paper-based ELISA. Angew Chem Int Ed Engl. **2010**;49:4771-4.

10. Tiwari S, Vinchurkar M, Rao VR, Garnier G. Zinc oxide nanorods functionalized paper for protein preconcentration in biodiagnostics. Sci Rep. **2017**;7:43905.

11. Wang S, Ge L, Song X, Yu J, Ge S, Huang J, et al. Paper-based chemiluminescence ELISA: lab-on-paper based on chitosan modified paper device and wax-screen-printing. Biosens Bioelectron. **2012**;31:212-8.

12. Nielsen K, Yu WL. Serological diagnosis of brucellosis. Prilozi. **2010**;31:65-89.

13. Vered O, Simon-Tuval T, Yagupsky P, Malul M, Cicurel A, Davidovitch N. The Price of a Neglected Zoonosis: Case-Control Study to Estimate Healthcare Utilization Costs of Human Brucellosis. PLoS One. **2015**;10:e0145086.

14. Hou H, Liu X, Peng Q. The advances in brucellosis vaccines. Vaccine. **2019**; 37:3981-8.

15. Verdiguel-Fernández L, Oropeza-Navarro R, Ortiz A, Robles-Pesina MG, Ramírez-Lezama J, Castañeda-Ramírez A, et al. Brucella melitensis omp31 Mutant Is Attenuated and Confers Protection Against Virulent Brucella melitensis Challenge in BALB/c Mice. J Microbiol Biotechnol. **2020**;30:497-504.

16. Gupta S, Singh D, Gupta M, Bhatnagar R. A combined subunit vaccine comprising BP26, Omp25 and L7/L12 against brucellosis. Pathog Dis. **2019**;

77:ftaa002.
17. Rezaei M, Rabbani-Khorasgani M, Zarkesh-Esfahani SH, Emamzadeh R, Abtahi H. Prediction of the Omp16 Epitopes for the Development of an Epitope-based Vaccine Against Brucellosis. Infect Disord Drug Targets. **2019**;19:36-45.
18. Degos C, Hysenaj L, Gonzalez-Espinoza G, Arce-Gorvel V, Gagnaire A, Papadopoulos A, et al. Omp25-dependent engagement of SLAMF1 by Brucella abortus in dendritic cells limits acute inflammation and favours bacterial persistence in vivo. Cell Microbiol. **2020**;22:e13164.
19. D'Annessa I, Di Leva FS, La Teana A, Novellino E, Limongelli V, Di Marino D. Bioinformatics and Biosimulations as Toolbox for Peptides and Peptidomimetics Design: Where Are We? Front Mol Biosci. **2020**;7:66.
20. Agyei D, Tsopmo A, Udenigwe CC. Bioinformatics and peptidomics approaches to the discovery and analysis of food-derived bioactive peptides. Anal Bioanal Chem. **2018**;410:3463-72.
21. Backert L, Kohlbacher O. Immunoinformatics and epitope prediction in the age of genomic medicine. Genome Med. **2015**;7:119.
22. Fu H, Song P, Wu Q, Zhao C, Pan P, Li X, et al. A paper-based microfluidic platform with shape-memory-polymer-actuated fluid valves for automated multi-step immunoassays. Microsyst Nanoeng. **2019**;5:50.
23. Pang B, Zhao C, Li L, Song X, Xu K, Wang J, et al. Development of a low-cost paper-based ELISA method for rapid Escherichia coli O157:H7 detection. Anal Biochem. **2018**; 542:58-62.

24. Cui H, Song W, Cao Z, Lu J. Simultaneous and sensitive detection of dual DNA targets via quantum dot-assembled amplification labels. Luminescence. **2016**; 31:281-7.

25. Tian Y, Zhang L, Wang L. DNA-Functionalized Plasmonic Nanomaterials for Optical Biosensing. Biotechnol J. **2020**;15:e1800741.

26. Marie M, Manoharan A, Kuchuk A, Ang S, Manasreh MO. Vertically grown zinc oxide nanorods functionalized with ferric oxide for in vivo and non-enzymatic glucose detection. Nanotechnology. **2018**;29:115501.

Supporting information

Table S1 Information of the patient

Order No.	Blood culture serial number	Pathogens
1	200707B0000035	Pseudomonas putida
2	200706B0000086	Aeromonas sobria
3	200705B0000069	Staphylococcus haemolyticus
4	200706B0000032	Escherichia coli
5	200704B0000100	Staphylococcus aureus
6	200708B0000072	Klebsiella pneumoniae
7	200708B0000168	Escherichia coli
8	200708B0000113	Staphylococcus saprophyticus
9	200708B0000051	Moraxella osloensis
10	200709B0000026	Staphylococcus hominis
11	200709B0000099	Raoultella ornithinolytica
12	200714B0000016	Escherichia coli
13	200713B0000052	Escherichia coli
14	200711b0000011	Candida parapsilosis
15	200715B0000042	Staphylococcus aureus
16	200715B0000014	Escherichia coli
17	200714B0000045	Staphylococcus epidermidis
18	200720B0000036	Klebsiella pneumoniae

19	200721B0000130	Streptococcus
20	200722B0000005	Pseudomonas aeruginosa
21	200722B0000074	Streptococcus constellatus
22	200726B0000056	Pseudomonas aeruginosa
23	200726B0000039	Rothia mucilaginos
24	200726B0000105	Enterococcus faecium
25	200726B0000040	Staphylococcus hominis
26	200725B0000017	Klebsiella pneumoniae
27	200727B0000068	Escherichia coli
28	200727B0000065	Staphylococcus aureus
29	200724B0000116	Escherichia coli
30	200728B0000078	Enterococcus faecium
31	200728B0000030	Escherichia coli
32	200727B0000128	Escherichia coli
33	200801B0000030	Enterococcus faecium
34	200802B0000001	Staphylococcus aureus
35	200801B0000108	Staphylococcus aureus
36	200803B0000007	Pseudomonas aeruginosa
37	200805B0000057	Staphylococcus aureus
38	200804B0000088	Streptococcus dysgalactiae
39	200804B0000051	Escherichia coli
40	200805B0000125	Escherichia coli

Table S2. The OMPs' Accession Numbers of Brucella. in NCBI Protein database

OMPs	Accession Numbers of *Brucella* spp.		
	B. melitensis	*B. abortus*	*B. suis*
BP26	AAB38523.1	AAO39773.1	KFJ31678.1
Omp16	AEF59023.1	AGI97133.1	AIB29945.1
Omp25	AEF59022.1	AFJ79953.1	AHN46339.1
Omp31	ACS50328.1	-	AAL27290.1
Omp2b	AMM72579.1	SUW28200.1	SUW48930.1

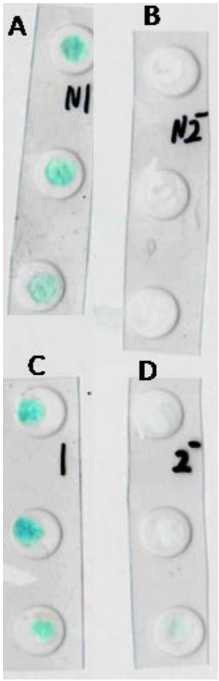

Figure S1 Results of p-ELISA. (A) Positive of nano-p-ELISA. (B) Negative of nano-p-ELISA. **(C)** Positive of tra-p-ELISA. (D) Negative of tra-p-ELISA.
