# Supplementary material for: Paper-based ELISA diagnosis technology for human brucellosis based on a multiepitope fusion protein
Source: PLoS Negl Trop Dis. 2021 Aug 17;15(8):e0009695. doi: 10.1371/journal.pntd.0009695 (PMC8396774; doi:10.1371/journal.pntd.0009695)
Supplement: Attachment — Submitted filename: PNTD-D-21-00330_R1(1)-seen.pdf [file pntd.0009695.s012.pdf]

PLOS Neglected Tropical Diseases

Paper-based ELISA diagnosis technology for human brucellosis based on a multiepitope fusion protein

--Manuscript Draft--

**Manuscript Number:**	PNTD-D-21-00330R1
**Full Title:**	Paper-based ELISA diagnosis technology for human brucellosis based on a multiepitope fusion protein
**Short Title:**	P-ELISA for diagnosing human brucellosis
**Article Type:**	Research Article
**Keywords:**	brucellosis; B cell epitope; nano-zinc oxide; p-ELISA; serological diagnosis
**Abstract:**	Background: Brucellosis, as a serious zoonotic infectious disease, has been recognized as a re-emerging disease in the developing countries worldwide. In china, the incidence of brucellosis is increasing each year, seriously threatening the health of humans as well as animal populations. Despite a quite number of diagnostic methods currently being used for brucellosis, innovative technologies are still needed for its rapid and accurate diagnosis, especially in area where traditional diagnostic is unavailable.<br>Methodology/Principal Findings: In this study, a total of 22 B cell linear epitopes were predicted from five Brucella outer membrane proteins (OMPs) using an immunoinformatic approach. These epitopes were then chemically synthesized, and with the method of indirect ELISA (iELISA), each of them displayed a certain degree of capability in identifying human brucellosis positive sera. Subsequently, a fusion protein consisting of the 22 predicted epitopes was prokaryotically expressed and used as diagnostic antigen in a newly established brucellosis testing method, nano-ZnO modified paper-based ELISA (nano-p-ELISA). According to the verifying test using a collection of sera collected from brucellosis and non-brucellosis patients, the sensitivity and specificity of multiepitope based nano-p-ELISA were 92.38% and 98.35% respectively. The positive predictive value was 98.26% and the negative predictive value was 91.67%. The multiepitope based fusion protein also displayed significantly higher specificity than Brucella lipopolysaccharide (LPS) antigen.<br>Conclusions: B cell epitopes are important candidates for serologically testing brucellosis. Multiepitope fusion protein based nano-p-ELISA displayed significantly sensitivity and specificity compared to Brucella LPS antigen. The strategy applied in this study will be helpful to develop rapid and accurate diagnostic method for brucellosis in human as well as animal populations.
**Additional Information:**	
**Question**	**Response**
**Financial Disclosure**<br><br>Enter a financial disclosure statement that describes the sources of funding for the work included in this submission. Review the submission guidelines for detailed requirements. View published research articles from *PLOS NTDs* for specific examples.<br><br>This statement is required for submission and **will appear in the published article** if the submission is accepted. Please make sure it is accurate.	This work was supported by the National Natural Science Foundation of China (Grant number 81802101 , received by Dehui Yin). The funders had no role in study design, data collection and analysis, decision to publish, or preparation of the manuscript.

Powered by Editorial Manager® and ProduXion Manager® from Aries Systems Corporation

**Unfunded studies**<br><br>Enter: *The author(s) received no specific funding for this work.*<br><br>**Funded studies**<br><br>Enter a statement with the following details:<br>• Initials of the authors who received each award<br>• Grant numbers awarded to each author<br>• The full name of each funder<br>• URL of each funder website<br>• Did the sponsors or funders play any role in the study design, data collection and analysis, decision to publish, or preparation of the manuscript?<br>• **NO** - Include this sentence at the end of your statement: *The funders had no role in study design, data collection and analysis, decision to publish, or preparation of the manuscript.*<br>• **YES** - Specify the role(s) played.<br><br>* typeset	
**Competing Interests**<br><br>Use the instructions below to enter a competing interest statement for this submission. On behalf of all authors, disclose any competing interests that could be perceived to bias this work—acknowledging all financial support and any other relevant financial or non-financial competing interests.<br><br>This statement **will appear in the published article** if the submission is accepted. Please make sure it is accurate. View published research articles from *PLOS NTDs* for specific examples.	The authors have declared that no competing interests exist.

**NO authors have competing interests**<br><br>Enter: *The authors have declared that no competing interests exist.*<br><br>**Authors with competing interests**<br><br>Enter competing interest details beginning with this statement:<br><br>*I have read the journal's policy and the authors of this manuscript have the following competing interests: [insert competing interests here]*<br><br>* typeset<br><br>This statement is required for submission and **will appear in the published article** if the submission is accepted. Please make sure it is accurate and that any funding sources listed in your Funding Information later in the submission form are also declared in your Financial Disclosure statement.	
**Data Availability**<br><br>Authors are required to make all data underlying the findings described fully available, without restriction, and from the time of publication. PLOS allows rare exceptions to address legal and ethical concerns. See the PLOS Data Policy and FAQ for detailed information.	Yes - all data are fully available without restriction

A Data Availability Statement describing where the data can be found is required at submission. Your answers to this question constitute the Data Availability Statement and **will be published in the article**, if accepted.<br><br>**Important:** Stating 'data available on request from the author' is not sufficient. If your data are only available upon request, select 'No' for the first question and explain your exceptional situation in the text box.<br><br>Do the authors confirm that all data underlying the findings described in their manuscript are fully available without restriction?	
**Describe where the data may be found in full sentences. If you are copying our sample text, replace any instances of XXX with the appropriate details.**<br><br>• If the data are **held or will be held in a public repository**, include URLs, accession numbers or DOIs. If this information will only be available after acceptance, indicate this by ticking the box below. For example: *All XXX files are available from the XXX database (accession number(s) XXX, XXX.*).<br>• If the data are all contained **within the manuscript and/or Supporting Information files**, enter the following: *All relevant data are within the manuscript and its Supporting Information files.*<br>• If neither of these applies but you are able to provide **details of access elsewhere**, with or without limitations, please do so. For example:	All relevant data are within the manuscript and its Supporting Information files.

Powered by Editorial Manager® and ProduXion Manager® from Aries Systems Corporation

Data cannot be shared publicly because of [XXX]. Data are available from the XXX Institutional Data Access / Ethics Committee (contact via XXX) for researchers who meet the criteria for access to confidential data.<br><br>*The data underlying the results presented in the study are available from (include the name of the third party and contact information or URL).*<br>• This text is appropriate if the data are owned by a third party and authors do not have permission to share the data.<br><br>* typeset	
Additional data availability information:	

Powered by Editorial Manager® and ProduXion Manager® from Aries Systems Corporation

Manuscript

Click here to access/download;Manuscript;Revised manuscript -clean version.doc

Paper-based ELISA diagnosis technology for human brucellosis based on a multiepitope fusion protein

Running head: P-ELISA for diagnosing human brucellosis

Dehui Yin[1#], Qiongqiong Bai[1#], Xiling Wu[1], Han Li[2], Jihong Shao[1], Mingjun Sun[3*],Hai Jiang[4*],Jingpeng Zhang[1*]

[1] Key Lab of Environment and Health, School of Public Health, Xuzhou Medical University, Xuzhou, 221004, China

[2]Department of Infection Control, the First Hospital of Jilin University, Changchun, 130021, China

[3]Laboratory of Zoonoses, China Animal Health and Epidemiology Center, Qingdao, 266032, China

[4]State Key Laboratory for Infectious Disease Prevention and Control, Collaborative Innovation Center for Diagnosis and Treatment of Infectious Diseases, National Institute for Communicable Disease Control and Prevention, Chinese Center for Disease Control and Prevention, Beijing, China

# These authors contributed equally to this work

*Correspondence:

Laboratory of Zoonoses, China Animal Health and Epidemiology Center, No.369 Nanjing Road, Qingdao, 266032, China. E-mail: sunmingjun@cahec.cn

State Key Laboratory for Infectious Disease Prevention and Control, Chinese Center for Disease Control and Prevention, No. 155 Changbai Road, Beijing, 102206, China.

E-mail: jianghai@icdc.cn.
School of Public Health, Xuzhou Medical University, No. 129 Tongshan Road, Xuzhou, 221004, China. E-mail: xiaopangpeng@126.com

Abstract

Background: Brucellosis, as a serious zoonotic infectious disease, has been recognized as a re-emerging disease in the developing countries worldwide. In china, the incidence of brucellosis is increasing each year, seriously threatening the health of humans as well as animal populations. Despite a quite number of diagnostic methods currently being used for brucellosis, innovative technologies are still needed for its rapid and accurate diagnosis, especially in area where traditional diagnostic is unavailable.

Methodology/Principal Findings: In this study, a total of 22 B cell linear epitopes were predicted from five *Brucella* outer membrane proteins (OMPs) using an immunoinformatic approach. These epitopes were then chemically synthesized, and with the method of indirect ELISA (iELISA), each of them displayed a certain degree of capability in identifying human brucellosis positive sera. Subsequently, a fusion protein consisting of the 22 predicted epitopes was prokaryotically expressed and used as diagnostic antigen in a newly established brucellosis testing method, nano-ZnO modified paper-based ELISA (nano-p-ELISA). According to the verifying test using a collection of sera collected from brucellosis and non-brucellosis patients, the sensitivity and specificity of multiepitope based nano-p-ELISA were 92.38% and 98.35% respectively. The positive predictive value was 98.26% and the negative predictive value was 91.67%. The multiepitope based fusion protein also displayed significantly higher specificity than *Brucella* lipopolysaccharide (LPS) antigen.

Conclusions: B cell epitopes are important candidates for serologically testing

brucellosis. Multiepitope fusion protein based nano-p-ELISA displayed significantly sensitivity and specificity compared to *Brucella* LPS antigen. The strategy applied in this study will be helpful to develop rapid and accurate diagnostic method for brucellosis in human as well as animal populations.

Keywords: brucellosis; B cell epitope; nano-zinc oxide; p-ELISA; serological diagnosis

Author Summary

Brucellosis is one of the most important zoonosis in the world and has caused tremendous economic losses in agriculture and animal husbandry in many countries. Developing rapid, sensitive and specific diagnostic methods is very important for early detection and treatment of brucellosis patients. In this study, a novel diagnostic technique, nano-ZnO modified paper ELISA, was established. The antigen used in this technique was a fusion protein containing multiple B cell epitopes, which were predicted from *Brucella* major outer membrane proteins such as Bp26, Omp31, Omp16, Omp2b and Omp25. Comparing to traditional LPS antigen, this multiepitope based antigen displayed considerably higher sensitivity and higher specificity in laboratory. With the strategy described in this paper, more efficient epitopes and protein antigen can be identified in the future. Currently, LPS antigen is only prepared from live *Brucella*, while protein antigen can be produced in large quantities in prokaryotic expression system. In addition to nano-p-ELISA, this protein antigen can also be used for development other methods such as fluorescent polarization assay (FPA) and immunochromatographic assay (ICA) to meet the varied demand for

brucellosis testing.

Introduction

Brucellosis is a reemerging zoonotic infectious disease. It not only seriously threatens the health of the people but also causes huge economic losses to animal husbandry industry. In human, brucellosis often manifests multiple symptoms and a long course of disease. So it is often misdiagnosed and causes increased cost of treatment and waste of medical resources [1]. Therefore, a rapid and accurate testing technology is very important for brucellosis diagnosis and subsequent treatment.

Currently, diagnostic methods for brucellosis include bacterial isolation, specific antibody detection and amplification of specific DNA fragments(PCR or qPCR) [2,3]. Bacterial isolation needs biosafety level 3 laboratory, and usually takes several weeks[4]. PCR or qPCR are fast and having higher sensitivity and specificity, but nucleic acid contamination often causes false positive result so that expensive facilities must be needed to guarantee the accuracy of testing [5]. On the other hand, antibody detection is the most popularly used method for testing brucellosis as they are easy to handle and suitable for most laboratories. There are several serological methods popularly used for antibody detection, including the agglutination test, complement fixation test (CFT), enzyme-linked immunosorbent assay (ELISA), immunochromatographic assay (ICA) and fluorescence polarization assay (FPA)[6]. All these methods are based on detecting antibody targeting for lipopolysaccharide (LPS). Although LPS is a major immunogen arousing high level of antibody titer, it contains the common epitope with other gram negative bacillus like *Yersinia* O:9 and *Escherichia* O:157, which greatly reduces its specificity in testing brucellosis. Moreover, as LPS is obtained only through culturing *Brucella* in high-level biosafety

facilities, it is not available to most of diagnostic kit manufacturers. Thus, seeking for new antigen to replace LPS is critical for developing easily available brucellosis testing kits.

In our previous study, the out membrane proteins such as Bp26 and Omp31 demonstrated considerable efficacy in detecting human brucellosis sera[7]. In this study, the B cell epitopes from five out membrane proteins were predicted and synthesized for the purpose of designing a more effective antigen. Paper-based enzyme-linked immunosorbent assay (p-ELISA) is an emerging technology. Due to the small reagent required and special equipment independence, it has attracted increasing attention from diagnostic reagent developers[8-10]. Here, using a prokaryotically expressed protein consisting of multiple B cell epitopes, a nano-ZnO modified p-ELISA (nano-p-ELISA) was established. The efficiency of this new method in detecting human brucellosis was evaluated against a collection of human sera. Hopefully, the epitope based protein can be applied in development of other fast and low cost diagnostic methods for brucellosis.

Methods

Human serum samples

121 human brucellosis sera were gifted by the School of Public Health of Jilin University. 90 negative control sera were collected by Infection Department of the

*sera from healthy individuals

First Clinical Hospital of Jilin University, including 50 *healthy sera and 40 patient sera confirmed by blood culture to be infected with other pathogens(S1 Table). All experiments were approved by the Animal Care and Ethics Committee of Xuzhou

Medical University.

B cell epitope prediction and synthesis

The amino acid sequences of *Brucella* out membrane protein Bp26, Omp2b, Omp16, Omp25 and Omp31 were download from NCBI website (https://www.ncbi.nlm.nih.gov/protein/). *Brucella* species and protein accession numbers were listed in supplementary S2 Table. The conserved amino acid sequences of these proteins were used to predict B cell epitopes using BepiPred tool in IEDB (http://tools.iedb.org/bcell/). Prediction threshold is 0.350(default value), above this threshold is possible epitope. Peptides longer than 6 amino acids were assumed as effective epitope and selected. Each of selected B cell epitope was chemically synthesized and coupled to keyhole limpet hemocyanin (KLH) in Sangon Biotech Company (Shanghai, China). The purity of each polypeptide-KLH was more than 90%.

Evaluation of B cell epitopes

121 human brucellosis positive sera were used to verify the diagnostic effect of predicted epitopes by indirect ELISA (iELISA). Each peptide-KLH was diluted with carbonate buffer (pH = 9.6) to final concentrations of 30 μg/mL. 100 μL of peptide-KLH was added to 96-well plate (Corning, USA) and incubated overnight at 4°C. 300 μL blocking solution (5% skimmed milk in PBS) was then added to plate and incubated at 37°C for 1 h. After washing 3 times with PBST, 100 μL of 1:400 diluted serum was added and incubated at 37℃ for 1 h. After washing 3 times with PBST, 1:5000 diluted HRP-labeled protein G (Thermo, USA) was added to plate and

incubated at room temperature for 30 min. In the coloring step, 100 μL of TMB substrate solution was added to each well and incubated for 15 min at room temperature. Coloring was terminated by adding 50 μL of stopping solution (2 M H_2SO_4). The optical density was measured at 450 nm (OD_{450}) using ELISA plate reader (BioTek, USA). At the same time, KLH (30 μg/mL, sigma) and lipopolysaccharide (LPS, 1 μg/mL provided by China Animal Health and Epidemiology Center) were used as controls in this experiment.

Fusion protein preparation and verification

Selected peptides were concatenated together and adjacent peptides were connected by a 'GGGS' linker(Fig S1). The DNA fragment corresponding to full length of concatenated peptides was synthesized and cloned into the expression vector pET-21a(+). Fusion protein containing concatenated peptides was expressed and purified from *E. coli* BL21(DE3) cells according to the optimized procedures (Sangon Biotech). The specific steps are described below.

After transferring the recombinant plasmid into BL21(DE3), 800 μL of nonresistant LB medium was added, followed by incubation at 37°C for 45 min and centrifugation at 5000 rpm for 3 min. Most of the supernatant was discarded (leave approximately 100-150 μL), the bacteria were resuspended, the LB plate with corresponding resistance was selected, and it was coated. After air-drying, it was inverted and cultured overnight in a 37°C incubator. The monoclonal colonies on the plate were chosen, placed into 10 mL of LB liquid medium and incubated at 37°C and 200 rpm. The cultured bacterial solution was transferred to 750 mL of LB liquid

medium at 37°C and 200 rpm, cultured to OD_{600}=0.6-0.8 with IPTG (0.5 mM) at 16°C and induced overnight. Then, the cells were centrifuged at 6000 rpm for 5 min, the supernatant was discarded, and the bacteria were collected. Bacteria were blown away with 20-30 mL 10 mM Tris-HCl (pH = 8.0) solution and ultrasonically broken (500 W, 60 times, 10 s each time, 15 s interval). After sonication, 100 μL of the bacterial suspension was centrifuged at 12000 rpm for 10 min, and 50 μL of supernatant was transferred to another EP tube. After the supernatant was removed, the precipitate was blown away with 50 μL of 10 mM Tris-HCl (pH = 8.0) solution. SDS-PAGE and Western blotting were used to detect protein expression. A nickel column (Ni Sepharose 6 Fast Flow, GE Healthcare) for affinity chromatography was used for protein purification. *Taking 5 mL of Ni-NTA, the equilibrium column was washed with 5 times the column bed volume of binding buffer at a flow rate of 5 mL/min. The crude protein was incubated with the equilibrated column packing for 1 h; the incubated product was loaded onto the column and the effluent liquid was collected; the equilibrium column was washed with binding buffer; the column was washed with washing buffer, and the effluent liquid was collected; with the column was eluted with elution buffer, and the effluent liquid was collected; and the crude protein was treated, washed with effluent and eluted with effluent separately, followed by sample preparation and, SDS-PAGE and WB detection. The concentrated protein was divided into 1 mL/tube and stored at -80°C.

*Please rephrase the sentence

Evaluation of the diagnostic effect of fusion protein

Diagnostic effect of the fusion protein was evaluated according to the iELISA

method described in **Evaluation of B cell epitopes section**. In this experiment, 1 μg fusion protein was coated to each well in 96-well plate, while other conditions were not changed.

Establishment of nano-p-ELISA

ZnO nanorods were synthesized on Whatman No. 1 filter paper by a hydrothermal method[11]. Whatman filter paper was soaked in 100 mM zinc acetate solution for 60 s and then annealed at 100 ℃ for 1 h to form a seed layer. Then, the filter paper was transferred to a hydrothermal reaction vessel containing 100 mM hexamethylenetetramine and $Zn(NO_3)_2.6H_2O$. Whatman filter paper was left at 90 ℃ for 5 h for formation of ZnO nanorods. Filter paper was then immersed in an hydrous toluene solution with 1% APTES for 5 min, dried at 100 ℃ for 15 min and then silanized. Scanning electron microscopy (SEM, JSM-7500F), X-ray diffraction (XRD, Bruker D8) and X-ray photoelectron spectroscopy (XPS, Escalable250Xi) were used to evaluate the nanorods structure on the surface of the paper. Nano-ZnO modified Whatman filter paper was punched into circular pieces with a diameter of 10 mm and A4 plastic packaging paper was punched into small holes with a diameter of 6 mm. The 10 mm circle paper was placed in the center of the 6 mm holes of the plastic packaging paper, fixed by a plastic packaging machine, and cut into small strips for further use.

Evaluation of the diagnostic effect of nano-p-ELISA

Five microliters of fusion protein solution (30 μg/mL in PBS) was placed in each well, followed by incubation at room temperature for 30 min, washing with 20 μL of

deionized water 3 times, and blocking with 20 μL of 5% skimmed milk powder at room temperature for 15 min. After washing 3 times with PBST, 5 μL of serum was added (diluted with 1:400) to the paper and incubated for 30 min. After washing 3 times with PBST, 5 μL of HRP labeled protein G was added (diluted with 1:8000), followed by incubation at room temperature for 210 s. After washing another 3 times, 5 μL of TMB substrate solution was added and incubated for 10 min. HP Laser Jet Pro MFP M227 was used to scan the paper. Image J software was used to carry out intensity analysis on developed color. To compare with the nano-p-ELISA method, the traditional p-ELISA (tra-p-ELISA) method was also performed according to literature[12]. Five microliters of chitosan dissolved in deionized water (0.25 mg/mL) was placed onto Whatman No.1 filter paper and air dried at room temperature. Then, 5 μL of 2.5% glutaraldehyde solution was added to the paper and incubated at room temperature for 2 h. The remaining steps are same as described in nano-p-ELISA.

Statistical analysis

Dot plot and receiver operating characteristic (ROC) curve analyses were performed using GraphPad Prism version 6.05. The gray intensity were determined by Student's t-test (unpaired t-test). *P*-values< 0.05 were considered to be significantly different.

Results

B cell epitope prediction and evaluation

From five *Brucella* antigen proteins, BP26, Omp16, Omp25, Omp31 and Omp2b predicted 14, 8, 14, 12, 19 epitopes respectively, and finally a total of 22 epitopes

were selected(Table 1). The length of these peptides ranged from 9 to 28 amino acid. The 22 polypeptide epitopes were synthesized and coupled to KLH. The results of iELISA showed that these epitopes have different ability in identifying human brucellosis positive sera(Fig 1). Average OD_{450} value of KLH plus 3 times Standard Deviation (SD) was used as threshold to distinguish positive and negative samples, here the threshold value being calculated as 0.5178, the epitope with the highest capability was P19266-6, as from 121 human brucellosis serum samples, 79 samples were detected as above the threshold. The other six epitopes (P19266-5, 12, 13, 15, 16 and 18) displayed medium capability with 37, 43, 44, 33, 24 and 33 samples were identified respectively. The remaining epitopes showed only limited capability, with

Please add space between 'detected' and '(Table 1)'

no more than 20 positive samples being detected(table 1).

Table 1 Information about 22 predicted B cell epitopes

**Protein**	**Epitope (amino acid sequence)**	**Start-end position**	**Length**	**Peptide ID**	**Number of positive sera recognized (n=121)**
	AFAQENQMTTQPARIAV	26-42	17	P19266-1	18
	KAGIEDRDLQTGGIN	88-102	15	P19266-2	17
	QPIYVYPDDKNNLKEPTITGY	104-124	21	P19266-3	17
BP26	GVNQGGDLNLVNDNPSAVIN	151-170	20	P19266-4	19
	LSRPPMPMP	204-212	9	P19266-5	37
	AAAPDNSVPIAAGENSYNVSVNVVFE	223-248	26	P19266-6	79
	SGAQAADAIVAPEPEAVEY	31-49	19	P19266-7	10
	DVKGGDDVYSGTDRNGWDK	79-97	19	P19266-8	12
Omp2b	NNSGVDGKYGNETSSGTV	129-146	18	P19266-9	10
	TVTPEVSYTKFGGEWKNTVAEDNAWGGI	341-368	28	P19266-10	11
	AAAPGSSQDFTV	44-55	12	P19266-11	1
Omp16	SRGVPTNRMRTISYGNERPVAVCD	125-148	24	P19266-12	42
Omp25	GRAKLENRTNGGTS	56-69	14	P19266-13	44

	GNPVQTTGETQ	115-125	11	P19266-14	1
	GGIKNSLRIGGEESSKSKTQT	154-174	21	P19266-15	33
	GWTVGAGIEYAA	175-186	12	P19266-16	24
	TDYGKKNFGLNDLDTRGSFKT NDIR	199-223	25	P19266-17	6
	VSEPSAPTAAPVDTFSWTGGYI GINA	24-49	26	P19266-18	33
	GKFKHPFSSFDKEDNEQVSGSL	53-75	23	P19266-19	8
Omp31	TGSISAGASGLEGKAE	112-127	16	P19266-20	7
	GDDASALHTWSDKTKAGWTL GAGAEYA	168-194	27	P19266-21	4
	DLGKRNLVD	209-217	9	P19266-22	8

Fig 1. Dotplot result of 22 epitopes in identifying 121 human brucellosis positive serum by iELISA

Fusion protein preparation and its diagnostic effect

As each of 22 predicted epitopes demonstrated some extent discerning capability for brucellosis sera, all these epitopes were included for constructing a fusion protein. SDS-PAGE showed that the molecular weight of prokaryotically expressed fusion protein was approximately 66 kDa (Figs 2A and 2B). Western blotting using anti-his tag antibody showed the same result (Fig 2C). Further mass spectrometry verified that the fusion protein was correctly expressed.

The diagnostic effect of the purified fusion protein was verified using121 human brucellosis sera and 90 control sera. For the fusion protein, the area under the ROC curve was 0.9877 (95% CI: 0.9758 to 0.9996), while the area under the ROC curve for LPS was 0.9174 (95% CI: 0.8796 to 0.9552) (Fig 3**)**, indicating that fusion protein has higher diagnostic effectiveness than LPS. The optimal cutoff value was also calculated by the Youden index, under which the positive predictive value (PPV) and

negative predictive value (NPV) of fusion protein were higher than those of LPS(Table 2). Under the cutoff value, 3 negative samples were misdiagnosed as positive with fusion protein. However, 20 negative samples was misdiagnosed as positive with LPS. This data indicated that the fusion protein had better specificity than LPS.

Table 2. Positive and negative predictive values of the test calculated for different cutoff values

Cutoff value	Positive		Negative		PPV (%)	NPV (%)
	TP	FN	TN	FP		
≥0.470 (fusion protein)	117	4	87	3	95.90	95.51
≥0.4095 (LPS)	115	6	70	20	85.19	92.10
≥50.98 (nano-p-ELISA)	113	8	88	2	98.26	91.67
≥45.66 (tra-p-ELISA)	113	8	87	3	97.41	91.58

TP, true positives; TN, true negatives; FP, false positives; FN, false negatives; PPV, positive predictive value (TP/TP+FP)×100; NPV, negative predictive value (TN/TN+FN) ×100.

Fig 2. SDS-PAGE and Western blot analysis of fusion protein. (A)SDS-PAGE result of fusion protein in the process of purification (M, marker; lane1, loading solution; lane2, flow-through solution; lane3-4, 20 mM imidazole elution fraction; lane5, 50 mM imidazole elution fraction; lane6, 500 mM imidazole elution fraction). (B) SDS-PAGE result of purified fusion protein (M, marker; lane1, purified protein). (C)

Western blot result of purified fusion protein (M, marker; lane1, purified protein).

Fig 3. Comparison of fusion protein and LPS in detecting human brucellosis positive and negative sera. (A) Dotplot result of iELISA assay with fusion protein. (B) ROC analysis of iELISA assay with fusion protein.(C) Dotplot result of iELISA assay with LPS antigen. (D) ROC analysis of iELISA assay with LPS antigen.

Characterization of nano-ZnO

XRD results showed that the main composition of nano-ZnO was successfully formed(Fig 4A). Scanning electron microscopy showed the shape of nano-ZnO on the surface of Whatman filter paper (Fig 4B). XPS showed that the concentration of Zn atoms was 40.79% and the concentration of oxygen atoms was 59.21%, which further indicated that the nano-crystal was composed of ZnO.

Fig 4. Characterization of prepared nano-ZnO. (A) XRD analysis of nano-ZnO. (B) The shape of nano-ZnO acquired by scanning electron microscope.

Evaluation of the diagnostic effect of nano-p-ELISA

Using 211 human brucellosis positive and negative sera, the ROC curve was obtained for nano-p-ELISA. Under the optimal cutoff value, 113 out of 121 positive samples were accurately diagnosed, and 88 out of the 90 negative samples were correctly identified. The positive predictive value of the nano-p-ELISA was 98.26%, and the negative predictive value was 91.67% (Table 2). There was a significant difference between the positive and negative samples($P<0.001$) (Fig 5A). The area

under the curve was 0.9900 (95% CI, 0.9816 to 0.9984), indicating that this method performed well in diagnosing human brucellosis. The optimal cutoff value was 50.98, under which the sensitivity of this method was 92.38% (95% CI, 0.8554 to 0.9665) and the specificity was 98.35% (95% CI, 0.9416 to 0.9980)(Fig 5B).

Correspondingly, the gray intensities and ROC curve of tra-p-ELISA demonstrated a similar performance as nano-p-ELISA(Figs 5C and 5D). Sensitivity and specificity analysis also supported this conclusion**(**Table 2**)**.

Fig 5. P-ELISA analysis of human serum samples. (A**)**Dotplotresult of the nano-p-ELISA assay. (B) ROC analysis of nano-p-ELISA assay results. (**C)** Dotplot result of tra-p-ELISA assay. (D) ROC analysis of tra-p-ELISA assay results.

Discussion

At present, the most of commercially available brucellosis detecting kits are based on the LPS which is polysaccharides compound on the surface of *Brucella*. Although single antigenic epitope of LPS can be chemically synthesized[13], under most circumstances, acquisition of LPS is only achieved by culturing live *Brucella* in high-level biosafety laboratories, which is an insurmountable obstacle for most diagnostics manufacturers. Therefore, seeking for new candidate antigen to replace LPS is of great significance for developing brucellosis detecting kits which can be easily produced and available to all users.

According to literatures, many *Brucella* OMPs manifested strong capacity in arousing humoral immune response [14-16], and some of these proteins have also

been used for development of subunit vaccine against brucellosis[17,18]. In our previous study, some *Brucella* OMPs showed quite satisfactory result in diagnosing brucellosis, basically comparable to LPS antigen [7]. The data in this paper reflected that not only entire OMPs, the linear short peptides in these OMPs also maintain good antigenicity. Moreover, multiple epitopes were more effective than single epitopes in identifying human brucellosis positive sera. It seems that higher sensitivity of epitope-based protein antigen could be achieved by increasing the number of epitopes. But, more epitopes could imply lower specificity as the chance of including common epitopes with other pathogens can be simultaneously increased. Therefore, there is still a lot of trimming work to be done on this fusion protein, so that the most suitable epitopes are selected for future commercial use.

The immunoinformatic analysis is an emerging science that integrates life sciences, computer science and mathematics to accelerate the process for vaccine design, disease diagnosis and treatment, as well as diagnostic agent screening[19,20]. The online immunoinformatic tool (Bepipred Linear Epitope Prediction) used in this study were proved to be feasible in discerning viable B cell epitopes, as a quite number of predicted epitopes were subsequently confirmed to be effective in laboratory. On the other hand, there were still many predicted epitopes just showing limited diagnostic effects. Hopefully in the future, a combination of immunoinformatic tools can be set up and used to improve the efficiency of epitope prediction.

Fast and easy-to-perform are the most concerned features in developing novel brucellosis detection technologies, especially in the point-of-care testing of medical diagnosis, environmental surveillance and food safety analysis. P-ELISA has attracted the attention from many researchers due to its higher specificity, simplicity, rapidity, portability and low cost [22,23]. Currently, nanomaterial modified p-ELISA are more widely used as modification can increase the surface area of the paper[10, 24-26]. Although tra-p-ELISA and nano-p-ELISA demonstrated similar antigenic capability, the latter method is more suitable for rapid on-site detection as it uses fewer reagent and can be stored at room temperature for a long time.

In summary, using bioinformatic technology combined with nanomaterials, this performance has established a new type of brucellosis diagnostic technology, which has good potential application value. However, the brucellosis sera selected in this study were all clinically screened positive sera, and the number was limited. The diagnostic validity of this method requires a large number of clinical random samples for verification. Beside nano-p-ELISA, there are some other methods suitable for rapid on-site testing, such as FPA and ICA. Combining the multiepitope based antigen and well established fast testing methods, more brucellosis testing kits would be produced in the future to meet the varied demand for brucellosis testing.

Acknowledgments

We thank the School of Public Health of Jilin University for their gift of the brucellosis serum samples.

Competing interests

The authors declare that they have no competing interests

Funding

This work was supported by Young Scientists Fund of the National Natural Science Foundation of China (Grant number 81802101).

Author Contributions

Conceptualization: Dehui Yin, Mingjun Sun, Hai Jiang, Jingpeng Zhang.

Data Curation: Dehui Yin, Qiongqiong Bai, Xiling Wu, Han Li.

Formal Analysis: Dehui Yin, Qiongqiong Bai, Jihong Shao.

Funding Acquisition: Dehui Yin.

Investigation: Dehui Yin, Mingjun Sun.

Methodology: Dehui Yin, Qiongqiong Bai, Xiling Wu, Han Li, Jihong Shao, Mingjun Sun.

Project Administration: Dehui Yin, Mingjun Sun, Jingpeng Zhang.

Resources: Dehui Yin, Mingjun Sun, Han Li, Jingpeng Zhang.

Supervision: Dehui Yin, Mingjun Sun, Jingpeng Zhang.

Validation: Dehui Yin, Mingjun Sun.

Writing – Original Draft Preparation: Dehui Yin, Qiongqiong Bai.

Writing – Original Draft Preparation: Dehui Yin, Mingjun Sun, Hai Jiang, Jingpeng Zhang.

References

1. Olsen SC, Palmer MV. Advancement of knowledge of Brucella over the past 50 years. Vet Pathol.**2014**; 51:1076-89.

2. Pappas G, Papadimitriou P, Akritidis N, Christou L, Tsianos EV. The new global map of human brucellosis. Lancet Infect Dis.**2006**;6:91-9.

3. Dean AS, Crump L, Greter H, Schelling E, Zinsstag J. Global burden of human brucellosis: a systematic review of disease frequency. PLoSNegl Trop Dis.**2012**;6:e1865.

4. Araj GF. Update on laboratory diagnosis of human brucellosis. Int J Antimicrob Agents.**2010**;36Suppl1:12-7.

5. Zeybek H, Acikgoz ZC, Dal T, Durmaz R. Optimization and validation of a real-time polymerase chain reaction protocol for the diagnosis of human brucellosis. Folia Microbiol (Praha).**2020**;65:353-61.

6. Yagupsky P, Morata P, Colmenero JD. Laboratory Diagnosis of Human Brucellosis. ClinMicrobiol Rev.**2019**;33:e00073-19.

7. Bai Q, Li H, Wu X, Shao J, Sun M, Yin D. Comparative analysis of the main outer membrane proteins of Brucella in the diagnosis of brucellosis. Biochem Biophys Res Commun. **2021**;560:126-31.

8. Zhao Y, Zeng D, Yan C, Chen W, Ren J, Jiang Y, et al. Rapid and accurate detection of Escherichia coli O157:H7 in beef using microfluidic wax-printed paper-based ELISA. Analyst.**2020**;145:3106-15.

9. Kasetsirikul S, Umer M, Soda N, Sreejith KR, Shiddiky MJA, Nguyen NT. Detection of the SARS-CoV-2 humanized antibody with paper-based ELISA. Analyst.**2020**;145:7680-86.

10. Cheng CM, Martinez AW, Gong J, Mace CR, Phillips ST, Carrilho E, et al. Paper-based ELISA. AngewChemInt Ed Engl.**2010**;49:4771-4.

11. Tiwari S, Vinchurkar M, Rao VR, Garnier G. Zinc oxide nanorods functionalized paper for protein preconcentration in biodiagnostics. Sci Rep.**2017**;7:43905.

12. Wang S, Ge L, Song X, Yu J, Ge S, Huang J, et al. Paper-based chemiluminescence ELISA: lab-on-paper based on chitosan modified paper device and wax-screen-printing. BiosensBioelectron.**2012**;31:212-8.

13. McGiven J, Howells L, Duncombe L, Stack J, Ganesh NV, Guiard J, et al. Improved serodiagnosis of bovine brucellosis by novel synthetic oligosaccharide antigens representing the capping m epitope elements of Brucella O-polysaccharide. J Clin Microbiol. **2015**;53:1204-10.

14. Hou H, Liu X, Peng Q. The advances in brucellosis vaccines. Vaccine.**2019**; 37:3981-8.

15. Verdiguel-Fernández L, Oropeza-Navarro R, Ortiz A, Robles-Pesina MG, Ramírez-Lezama J, Castañeda-Ramírez A, et al. Brucella melitensis omp31 Mutant Is Attenuated and Confers Protection Against Virulent Brucella melitensis Challenge in

BALB/c Mice. J MicrobiolBiotechnol.**2020**;30:497-504.

16. Gupta S, Singh D, Gupta M, Bhatnagar R. A combined subunit vaccine comprising BP26, Omp25 and L7/L12 against brucellosis. Pathog Dis.**2019**; 77:ftaa002.

17. Rezaei M, Rabbani-Khorasgani M, Zarkesh-Esfahani SH, Emamzadeh R, Abtahi H. Prediction of the Omp16 Epitopes for the Development of an Epitope-based Vaccine Against Brucellosis. Infect Disord Drug Targets.**2019**;19:36-45.

18. Degos C, Hysenaj L, Gonzalez-Espinoza G, Arce-Gorvel V, Gagnaire A, Papadopoulos A, et al. Omp25-dependent engagement of SLAMF1 by Brucella abortus in dendritic cells limits acute inflammation and favours bacterial persistence in vivo. Cell Microbiol.**2020**;22:e13164.

19. D'Annessa I, Di Leva FS, La Teana A, Novellino E, Limongelli V, Di Marino D. Bioinformatics and Biosimulations as Toolbox for Peptides and Peptidomimetics Design: Where Are We? Front MolBiosci.**2020**;7:66.

20. Agyei D, Tsopmo A, Udenigwe CC. Bioinformatics and peptidomics approaches to the discovery and analysis of food-derived bioactive peptides. Anal Bioanal Chem.**2018**;410:3463-72.

21. Backert L, Kohlbacher O. Immunoinformatics and epitope prediction in the age of genomic medicine. Genome Med.**2015**;7:119.

22. Fu H, Song P, Wu Q, Zhao C, Pan P, Li X, et al. A paper-based microfluidic platform with shape-memory-polymer-actuated fluid valves for automated multi-step immunoassays. MicrosystNanoeng.**2019**;5:50.

23. Pang B, Zhao C, Li L, Song X, Xu K, Wang J, et al. Development of a low-cost paper-based ELISA method for rapid Escherichia coli O157:H7 detection. Anal Biochem.**2018**;542:58-62.

24. Cui H, Song W, Cao Z, Lu J. Simultaneous and sensitive detection of dual DNA targets via quantum dot-assembled amplification labels. Luminescence.**2016**; 31:281-7.

25. Tian Y, Zhang L, Wang L. DNA-Functionalized Plasmonic Nanomaterials for Optical Biosensing. Biotechnol J.**2020**;15:e1800741.

26. Marie M, Manoharan A, Kuchuk A, Ang S, Manasreh MO. Vertically grown zinc oxide nanorods functionalized with ferric oxide for in vivo and non-enzymatic glucose detection. Nanotechnology.**2018**;29:115501.

Supporting information

S1 Fig. Fig S1. Comprehensive sequence of amino acids of the fusion protein; the linkers are in red font.

S2 Fig. Fig S2. Results of p-ELISA. (A) Positive of nano-p-ELISA. (B) Negative of nano-p-ELISA. (**C)** Positive of tra-p-ELISA. (D) Negative of tra-p-ELISA.

S1 Table. Table S1. Information of the patient

S2 Table. Table S2. The OMPs' Accession Numbers of *Brucella* in NCBI Protein database

S1 Data. supporting data for figure 1.

S2 Data. supporting data for figure 3.

S3 Data. supporting data for figure 5.

S4 Data. supporting data for XPS

S5 Data. supporting data for figure 4A.

S1 File. Medical Ethics Review Report.

Figure 1

Click here to access/download;Figure;fig 1.tif

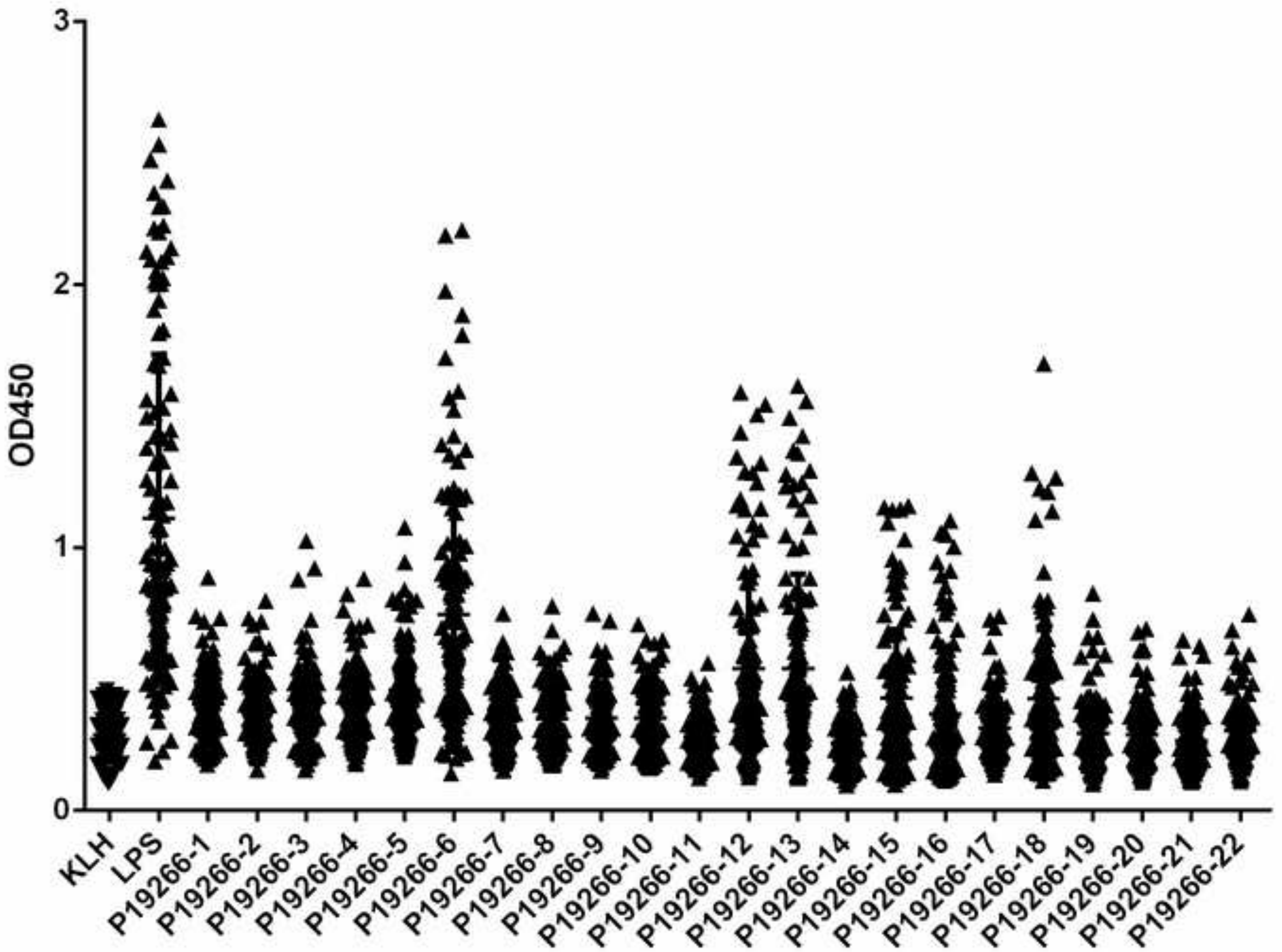

Figure 2

Click here to access/download;Figure;fig 2.tif

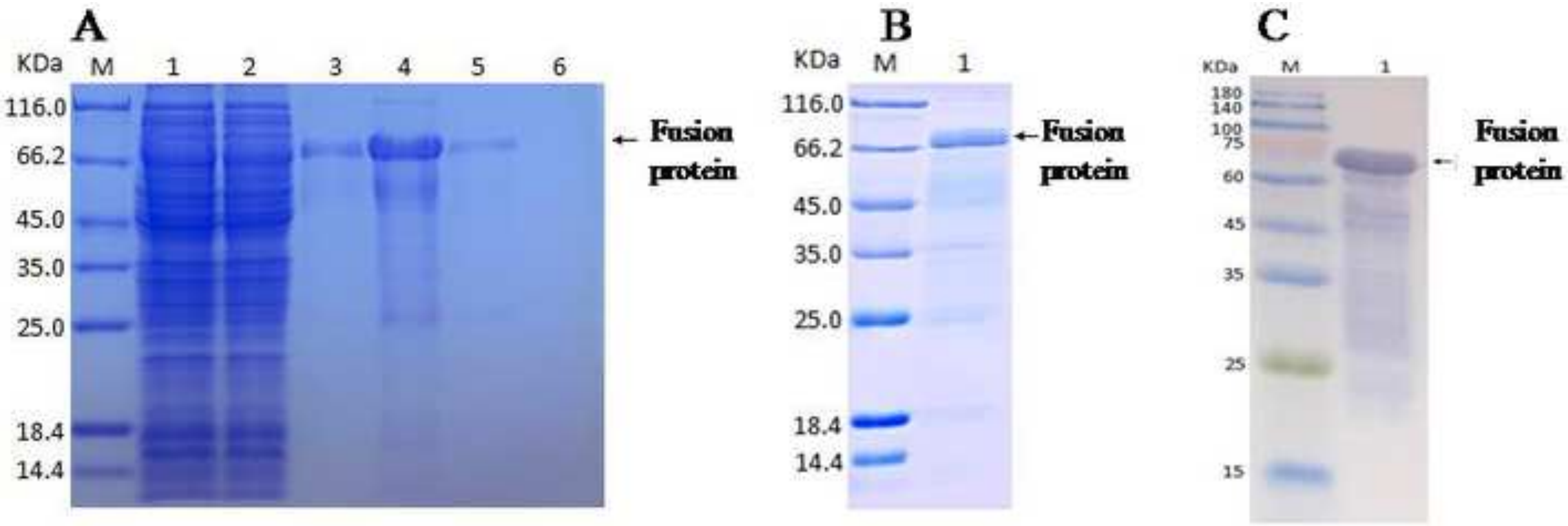

Figure 3 Click here to access/download;Figure;Fig 3.tif

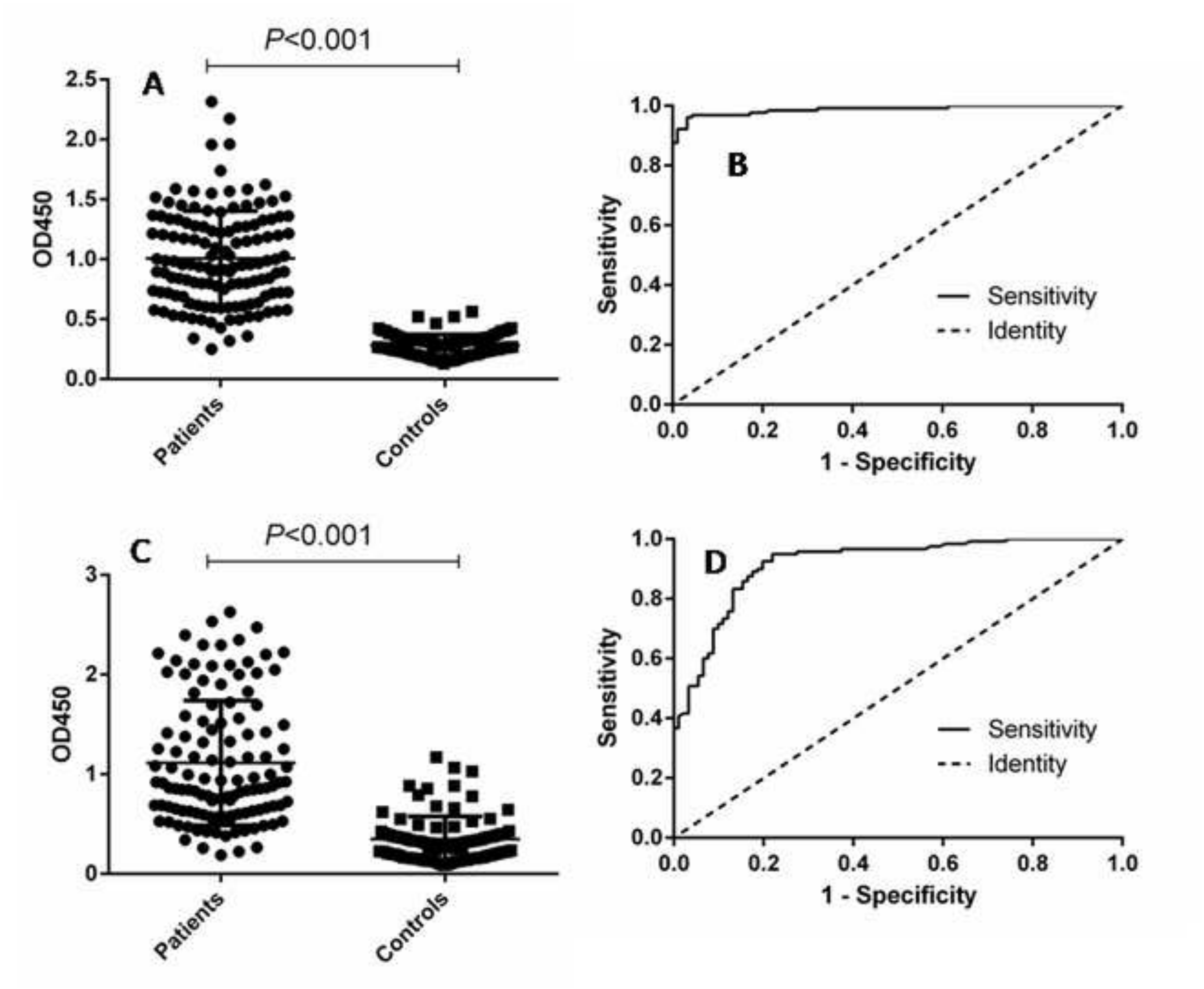

Figure 4

Click here to access/download;Figure;Fig 4.tif

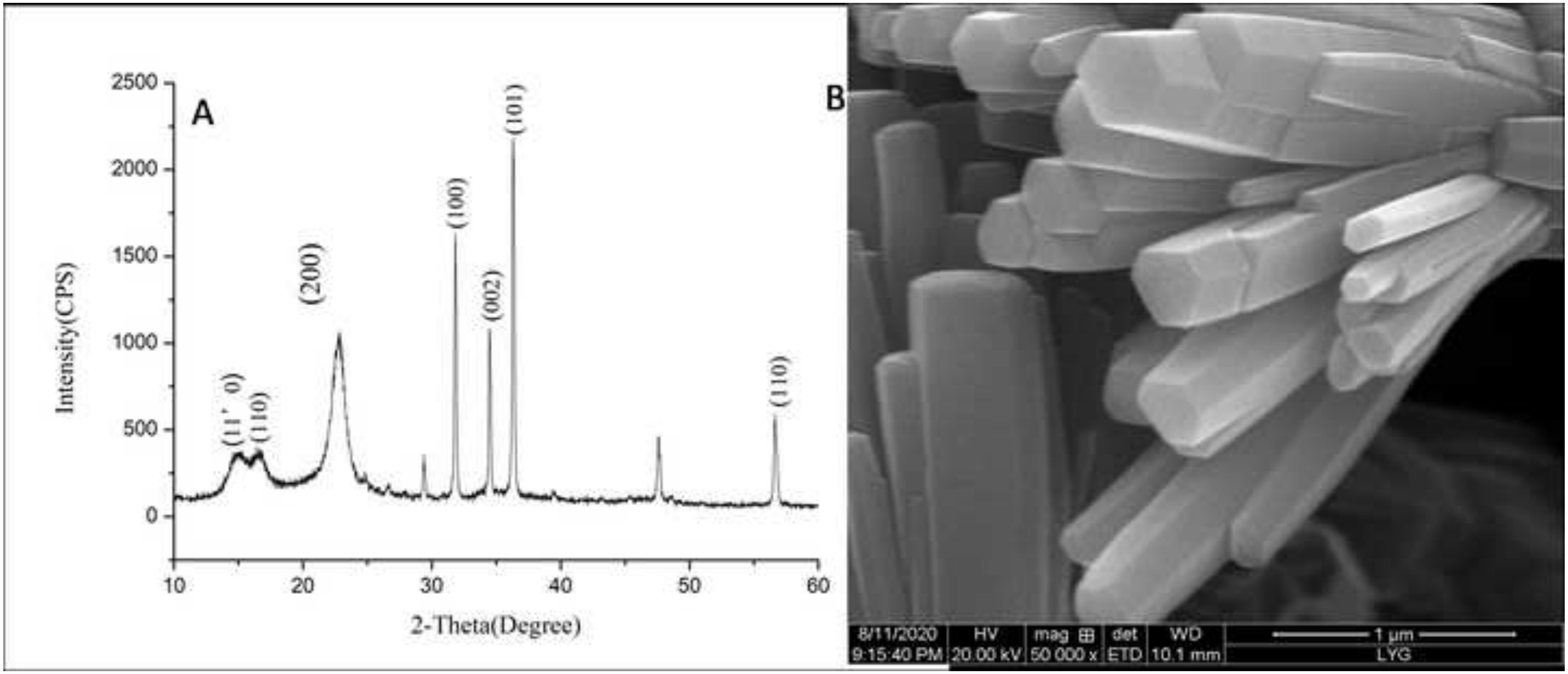

Figure 5

Click here to access/download;Figure;Fig 5.tif

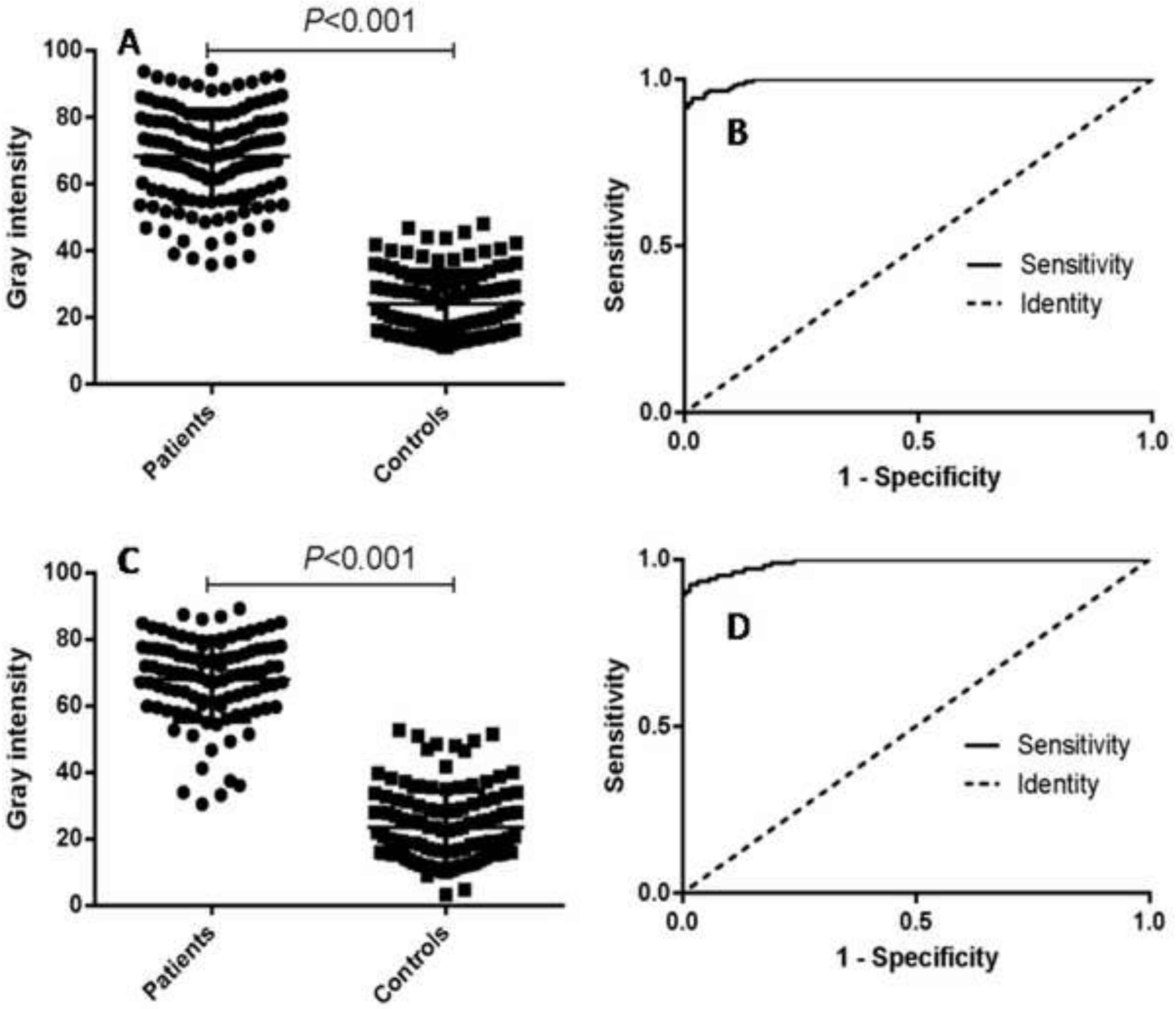

Click here to access/download

Supporting Information - Compressed/ZIP File Archive

Supporting information.zip

 Click here to access/download;Striking Image;Graphical Abstract Image.tif

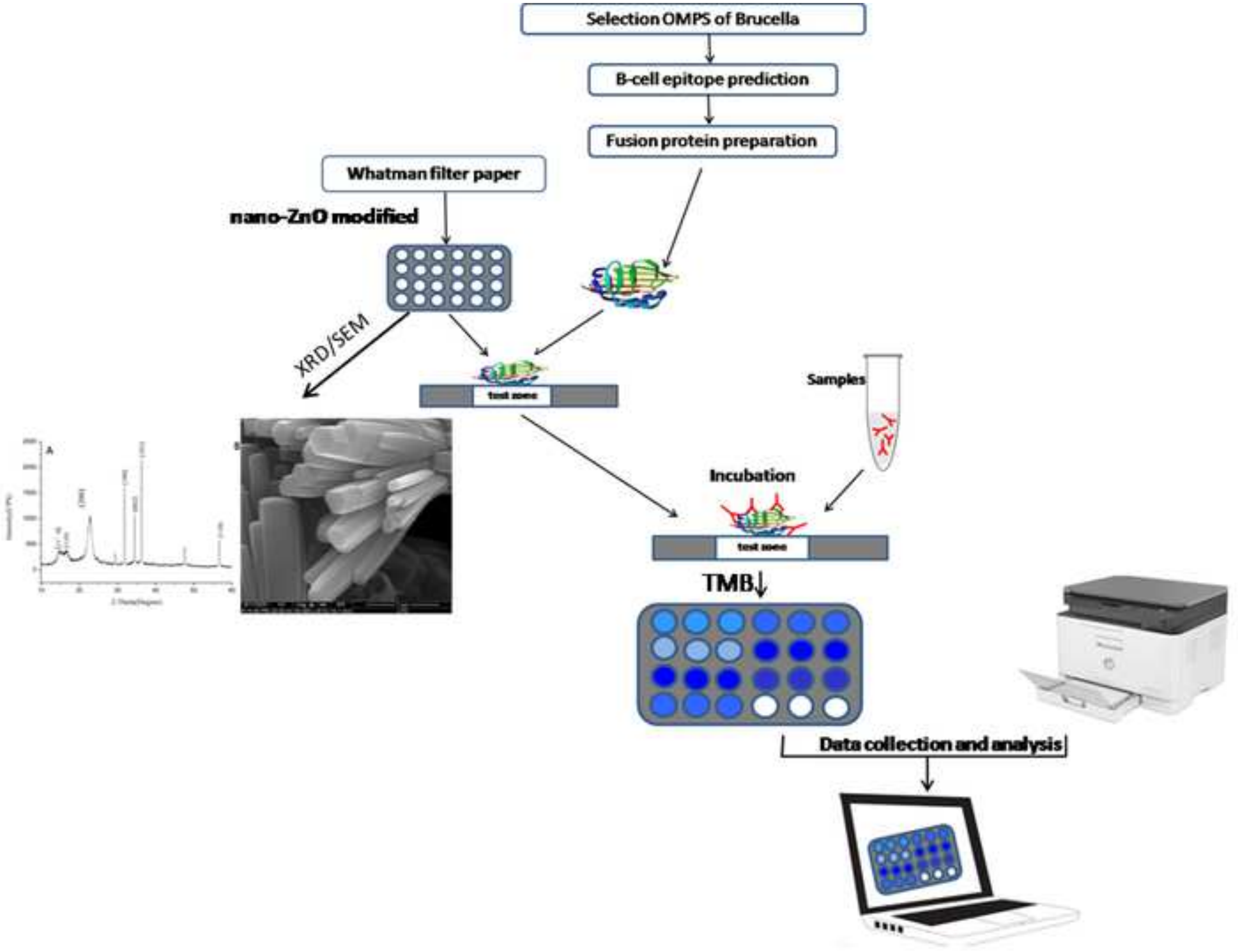

Dear Editors and Reviewers:

We really appreciate for your time and comments on our manuscript entitled "Paper-based ELISA diagnosis technology for human brucellosis based on a multiepitope fusion protein" (ID: PNTD-D-21-00330). Considering the reviewers' comments and the fact that English is not our native language, to make it more understandable, we consulted to native English speaker and made extensive revision on the main text, but the results and supporting materials such as tables and pictures were not changed. We hope that the massive text changes would not result in adverse effect that the revised manuscript could be considered as a new manuscript. The previous and revised manuscripts were provided for your consideration.

More attentions were paid to the sections reviewers most concerned, and modification has been made according to the reviewer's suggestion. To the reviewer's comments, our responses were listed as followings:

Methods
Reviewer #1:

1. Objectives and hypothesis are not clear in the manuscript. Methods need details and not matching with the results provided.

Response: The objective of this study is to provide a novel diagnostic antigen for brucellosis. To make the objective clearer, we reorganized the Introduction section, and added description about antigen used in current brucellosis testing methods. We hypothesized that the B cell epitopes predicted from *Brucella* out membrane proteins can be used to replace LPS for brucellosis serodiagnosis. So, in this study, a fusion protein containing multiepitopes were produced ant its efficacy in diagnosing human

brucellosis was evaluated with ELISA method. At the same time, the Method sand Results sections were carefully revised and more details were added. We believed that the results were much clearer in revised manuscript.

2. Section starting from L106 and 111 could be pooled together to make it comprehensive and easy to follow. The algorithm used to determine length and selection of sequence of peptides predicted and selected for the study are not mentioned. Also, the number of peptide epitopes predicted are missing. Were only 22 peptides predicted and used in screening by ELISA and for fusion protein preparation? Or 22 peptides were selected? The final length and details of sequence for fusion protein produced are missing in methods, as provided in table 1.

Response: Line 106 to line 111 has been revised in the new manuscript. The method used to determine length and selection of sequence of peptides were addedto the **Methods** Section. The prediction was mainly depended on the online software BepiPred (http://tools.iedb.org/bcell/) using default parameters. The output epitopes had different number of amino acid and some even contains just single amino acid. So an empirical value was used to choose the epitopes, here epitopes containing more than 6 amino acids were believed to have effective diagnostic capability. From five Omps, a total of 22 predicted peptides were found to be longer than 6 amino acids, and all of them were included in following verifying assay by ELISA. The method section has been modified so that the epitope prediction and selection step can be clearly described(Line 125). The final length and details of sequence for fusion

protein were also added, please see the **Supporting information (Fig S1)**.

3. L236-Describe the positive sera used for screening of peptides? Is it the reference sera or from 121 known positive sera? Were all sera reacted to all 22 peptides?

Response: We are very sorry that we did not describe the source of human sera clearly. We added the details in line 132. Reference sera for human brucellosis are not available presently. So, the 121 positive sera used for screening of peptides. According to the result, not all sera reacted strongly to these peptides, but each peptide can recognize a proper portion of these positive sera. We revised the data in Results section so that it can be clearly demonstrated(Table 1).

4. L213-nanomodified p-ELISA (nano-p-ELISA) method comparison using traditional p-ELISA is described, however the details for nano-p-ELISA is not mentioned in the manuscript.

Response: The method for nano-p-ELISA has been reorganized and described in Method Section (Line 167 to 213). The verifying data was displayed in Table 2. The nano-p-ELISA showed very similar efficacy in detecting human brucellosis as tra-p-ELISA, so we just described briefly the comparison result in Discussion section.

5. Sample size is sufficient and population is also clearly described. The statistical analysis support the findings. No concerns about ethical and regulatory requirements.

Response: Thank you very much for your comments. Actually, the study has been reviewed and approved by the Ethics Committee of Xuzhou Medical University, and related document was provided at the end of this cover letter.

Reviewer #2:

I would suggest for the authors to add the Figure on the schematic diagram of developed paper-based ELISA for human brucellosis using multiepitope fusion protein.

Response: Thank you very much for your suggestion, the schematic diagram of nano-ZnO modified paper-ELISA has been created, please see the Graphical Abstract Image.

Results

Reviewer #1:

1. Results of LPS antigen are shown in figure 3 C and D, but details of LPS antigen are missing in method section and not mentioned elsewhere in the manuscript.

Response: LPS antigen was provided by China Animal Health and Epidemiology Center. We had added the related information to Methods section, and please refer to the line 145 to 146.

2. L236-Describe the positive sera used for screening of peptides? Is it the reference sera or from 121 known positive sera? Were all sera reacted to all 22 peptides?

Response: We are very sorry that we did not describe the source of human sera clearly. We added the details in line 132. Reference sera for human brucellosis are not available presently. So, the 121 positive sera used for screening of peptides. According to the result, not all sera reacted strongly to these peptides, but each

peptide can recognize a proper portion of these positive sera. We revised the data in Results section so that it can be clearly demonstrated(Table 1)..

3. **Nanomodified p-ELISA (nano-p-ELISA) method comparison using traditional p-ELISA is unclear. In discussion L365, authors also mention comparison with traditional ELISA, please provide details.**

Response: Using 121 brucellosis positive sera and 90 negative sera, the sensitivity and specificity of nano-p-ELISA and tra-p-ELISA were evaluate and displayed in Table 2. As the two mehods showed very similar efficacy in detecting human brucellosis, we just described briefly the comparison result in Discussion section.

Reviewer #2:

Overall the results are clearly presented accept that Figure 1. I would suggest the authors to replace with better quality of the graph to show clearly results of iELISA of each peptide identification-positive brucellosis serum.

Response: Thank you for your suggestion, we have replaced the Figure 1 and with better quality of the graph.

Conclusions

Reviewer #1:

1. This section needs to be revised with relevance to findings of the manuscript.L340-341-should it be part of results?

Response: This section has been massively revised with relevance to the main findings. Some sentences apparently more suitable for Introduction or Result sections were removed from this section.

2. L345-346- need justification for the statement for cross-reactivity.

Response: After careful consideration, more accurate term to describe antigen's reactivity to negative sera is specificity. So, we deleted the word of *cross-reactivity* from the manuscript.

3.L357-359 should be part of introduction and not discussion.

Response: We made great change to the Discussion section, and these sentences has been deleted from the manuscript.

Reviewer #2:

1. The conclusions are supported the data presented and the authors have explained the public health relevance for human brucellosis by using the developed method (paper-based ELISA on multiepitope fusion protein)

Response: Thank you very much for your comments.

Editorial and Data Presentation Modifications?

Use this section for editorial suggestions as well as relatively minor modifications of existing data that would enhance clarity. If the only modifications needed are minor and/or editorial, you may wish to recommend "Minor Revision" or "Accept".

Reviewer #1:

Authors need to carefully review terminology used in the manuscript. The term "antigenicity" is misleading, when throughout the manuscript the fusion peptide was used as a target for detection of antibodies in human sera.

Response: Thank you very much for your comment. In this paper, we discussed mainly the capability of fusion peptides in serodiagnosis of human brucellosis. To avoid misunderstanding, we deleted the word of *antigenicity* from manuscript, and replace it with other words such as diagnostic effect.

Reviewer #2: Minor revision

Summary and General Comments
Reviewer #1: (No Response)

Reviewer #2:

1. This study is important to be published as the developed method will help the human brucellosis diagnosis.

Response: Thank you very much for your comment.

2. The study is novel and has shown the better performance in terms of sensitivity and specificity of the test.

Response: Thank you very much for your comment.

3. Please include the details of the ethical approval documents.

Response: Before we applied for this project from National Natural Science Foundation of China in 2018, the application has been reviewed and approved by the Ethics Committee of Xuzhou Medical University. We attached the original ethical documents in picture format as following. At the same time, we also provided the translated version for your consideration.

医学伦理审查报告

我校公共卫生学院拟开展“基于多表位融合蛋白的人布鲁氏菌病快速诊断技术研究”科研工作，该项目涉及布鲁氏菌病人血清标本的采集和检测，我校医学伦理委员会对该项目相关医学伦理学问题进行了审查。

项目信息：

项目名称：基于多表位融合蛋白的人布鲁氏菌病快速诊断技术研究

承担单位：徐州医科大学，项目负责人：殷德辉，职称：讲师

研究起止日期：2018 年 7 月至 2021 年 6 月

涉及人体研究的主要内容：

本项目拟以布鲁氏菌病病人血清为研究对象，利用生物信息学、融合蛋白、蛋白L和p-ELSIA技术，建立一种快速、灵敏的人布鲁氏菌病血清学诊断新技术，为针对性地开展人兽共患传染病-布鲁氏菌病的防治提供有价值的科学依据和新的策略，具有重要的现实意义。

审查评议意见：

经我校医学伦理委员会审议，该研究的实验设计和实施方案充分考虑了安全性和公平性原则，研究内容不构成对研究人员等人群的危害，研究内容和结果不存在利益冲突。

结论：

该研究中，实验内容符合医学伦理学要求，不存在潜在风险，同意该项研究的工作按计划进行。

徐州医科大学伦理委员会

2018 年 2 月 27 日

Translated version：

Medical Ethics Review Report

School of Public Health of our school intends to carry out the scientific research work of *Study on the rapid diagnosis technology of human brucellosis based on multiepitope fusion protein*. This work involves the collection and detection of serum samples from human. The Ethics Committee of Xuzhou Medical University carefully reviewed the related medical ethics issues about this project.

Project information:

Project name: Study on rapid diagnosis technology for human brucellosis based on

multiepitope fusion protein

Institute undertaking this project: Xuzhou Medical University

Project leader: Yin Dehui, Lecturer

Start and end dates: From January 2019 to December 2021

Main content involved in human research:

This project intends to use the serum of brucellosis patients together with bioinformatics, fusion protein, protein L and p-ELSIA technology to establish a rapid and sensitive new technology for the serological diagnosis of human brucellosis. It is of great significance for the prevention and treatment of the zoonotic infectious disease brucellosis in human, and also providing a valuable strategy for developing novel diagnostic technology for this disease.

Review comments:

After careful consideration by the Ethics Committee of Xuzhou Medical University, the research design and implementation fully comply with the principles of safety and fairness. The research content does not constitute any hazard factor to researchers and other populations, and there is no conflict of interest in the research content and results.

Conclusion:

In the study, the experimental content completely meets the requirements of medical ethics and there is no potential risk. Agreement is reached that this work can be carried out according to the plan.

Ethics Committee of Xuzhou Medical University

February 27, 2018

Figure Files:

While revising your submission, please upload your figure files to the Preflight Analysis and Conversion Engine (PACE) digital diagnostic tool, https://pacev2.apexcovantage.com. PACE helps ensure that figures meet PLOS requirements. To use PACE, you must first register as a user. Then, login and navigate to the UPLOAD tab, where you will find detailed instructions on how to use the tool. If you encounter any issues or have any questions when using PACE, please email us at figures@plos.org.

Response: We have uploaded all our figure files to the Preflight Analysis and Conversion Engine.

Data Requirements:

Please note that, as a condition of publication, PLOS' data policy requires that you make available all data used to draw the conclusions outlined in your manuscript. Data must be deposited in an appropriate repository, included within the body of the manuscript, or uploaded as supporting information. This includes all numerical values that were used to generate graphs, histograms etc.. For an example see here: http://www.plosbiology.org/article/info%3Adoi%2F10.1371%2Fjournal.pbio.1001908#s5.

Response: All data for our figure has been upload as supporting information.

Reproducibility:

To enhance the reproducibility of your results, we recommend that you deposit your laboratory protocols in protocols.io, where a protocol can be assigned its own identifier (DOI) such that it can be cited independently in the future. Additionally, PLOS ONE offers an option to publish peer-reviewed clinical study protocols. Read more information on sharing protocols at https://plos.org/protocols?utm_medium=editorial-email&utm_source=authorletters&utm_campaign=protocols

Response: Considering that this manuscript has been released as a preprint at Research Square (https://www.researchsquare.com/article/rs-291650/v1, DOI: 10.21203/rs.3.rs-291650/v1), we did not deposit it in protocols.io.

Once again, thank you very much for your comments and suggestions.

Best wishes,

Dehui Yin

Click here to access/download
Revised Article with Changes Highlighted
Revised manuscript- highlights.docx
