# Supplementary material for: Paper-based ELISA diagnosis technology for human brucellosis based on a multiepitope fusion protein
Source: PLoS Negl Trop Dis. 2021 Aug 17;15(8):e0009695. doi: 10.1371/journal.pntd.0009695 (PMC8396774; doi:10.1371/journal.pntd.0009695)
Supplement: Attachment — Submitted filename: cover letter 0627.docx [file pntd.0009695.s011.docx]

**Dear Editors and Reviewers:**

We really appreciate for your time and comments on our manuscript entitled "Paper-based ELISA diagnosis technology for human brucellosis based on a multiepitope fusion protein" (ID: PNTD-D-21-00330). Considering the reviewers' comments and the fact that English is not our native language, to make it more understandable, we consulted to native English speaker and made extensive revision on the main text, but the results and supporting materials such as tables and pictures were not changed. We hope that the massive text changes would not result in adverse effect that the revised manuscript could be considered as a new manuscript. The previous and revised manuscripts were provided for your consideration.

More attentions were paid to the sections reviewers most concerned, and modification has been made according to the reviewer’s suggestion. To the reviewer’s comments, our responses were listed as followings:

**Methods**
**Reviewer #1:**

1. **Objectives and hypothesis are not clear in the manuscript. Methods need details and not matching with the results provided.**

**Response:** The objective of this study is to provide a novel diagnostic antigen for brucellosis. To make the objective clearer, we reorganized the Introduction section, and added description about antigen used in current brucellosis testing methods. We hypothesized that the B cell epitopes predicted from *Brucella* out membrane proteins can be used to replace LPS for brucellosis serodiagnosis. So, in this study, a fusion protein containing multiepitopes were produced ant its efficacy in diagnosing human brucellosis was evaluated with ELISA method. At the same time, the Method sand Results sections were carefully revised and more details were added. We believed that the results were much clearer in revised manuscript.

2. **Section starting from L106 and 111 could be pooled together to make it comprehensive and easy to follow. The algorithm used to determine length and selection of sequence of peptides predicted and selected for the study are not mentioned. Also, the number of peptide epitopes predicted are missing. Were only 22 peptides predicted and used in screening by ELISA and for fusion protein preparation? Or 22 peptides were selected? The final length and details of sequence for fusion protein produced are missing in methods, as provided in table 1.**

**Response:** Line 106 to line 111 has been revised in the new manuscript. The method used to determine length and selection of sequence of peptides were addedto the **Methods** Section. The prediction was mainly depended on the online software BepiPred (http://tools.iedb.org/bcell/) using default parameters. The output epitopes had different number of amino acid and some even contains just single amino acid. So an empirical value was used to choose the epitopes, here epitopes containing more than 6 amino acids were believed to have effective diagnostic capability. From five Omps, a total of 22 predicted peptides were found to be longer than 6 amino acids, and all of them were included in following verifying assay by ELISA. The method section has been modified so that the epitope prediction and selection step can be clearly described(Line 125). The final length and details of sequence for fusion protein were also added, please see the **Supporting information (Fig S1)**.

**3. L236-Describe the positive sera used for screening of peptides? Is it the reference sera or from 121 known positive sera? Were all sera reacted to all 22 peptides?**

Response: We are very sorry that we did not describe the source of human sera clearly. We added the details in line 132. Reference sera for human brucellosis are not available presently. So, the 121 positive sera used for screening of peptides. According to the result, not all sera reacted strongly to these peptides, but each peptide can recognize a proper portion of these positive sera. We revised the data in Results section so that it can be clearly demonstrated(Table 1).

**4. L213-nanomodified p-ELISA (nano-p-ELISA) method comparison using traditional p-ELISA is described, however the details for nano-p-ELISA is not mentioned in the manuscript.**

**Response:** The method for nano-p-ELISA has been reorganized and described in Method Section (Line 167 to 213). The verifying data was displayed in Table 2. The nano-p-ELISA showed very similar efficacy in detecting human brucellosis as tra-p-ELISA, so we just described briefly the comparison result in Discussion section.

**5. Sample size is sufficient and population is also clearly described. The statistical analysis support the findings. No concerns about ethical and regulatory requirements.**

**Response**: Thank you very much for your comments. Actually, the study has been reviewed and approved by the Ethics Committee of Xuzhou Medical University, and related document was provided at the end of this cover letter.

**Reviewer #2:**

**I would suggest for the authors to add the Figure on the schematic diagram of developed paper-based ELISA for human brucellosis using multiepitope fusion protein.**

**Response:** Thank you very much for your suggestion, the schematic diagram of nano-ZnO modified paper-ELISA has been created, please see the Graphical Abstract Image.

**Results**
**Reviewer #1:**

1. **Results of LPS antigen are shown in figure 3 C and D, but details of LPS antigen are missing in method section and not mentioned elsewhere in the manuscript.**

**Response:** LPS antigen was provided by China Animal Health and Epidemiology Center. We had added the related information to Methods section, and please refer to the line 145 to 146.

**2. L236-Describe the positive sera used for screening of peptides? Is it the reference sera or from 121 known positive sera? Were all sera reacted to all 22 peptides?**

**Response:** We are very sorry that we did not describe the source of human sera clearly. We added the details in line 132. Reference sera for human brucellosis are not available presently. So, the 121 positive sera used for screening of peptides. According to the result, not all sera reacted strongly to these peptides, but each peptide can recognize a proper portion of these positive sera. We revised the data in Results section so that it can be clearly demonstrated(Table 1)..

3. **Nanomodified p-ELISA (nano-p-ELISA) method comparison using traditional p-ELISA is unclear. In discussion L365, authors also mention comparison with traditional ELISA, please provide details.**
   **Response:** Using 121 brucellosis positive sera and 90 negative sera, the sensitivity and specificity of nano-p-ELISA and tra-p-ELISA were evaluate and displayed in Table 2. As the two mehods showed very similar efficacy in detecting human brucellosis, we just described briefly the comparison result in Discussion section.

**Reviewer #2:**

**Overall the results are clearly presented accept that Figure 1. I would suggest the authors to replace with better quality of the graph to show clearly results of iELISA of each peptide identification-positive brucellosis serum.**

**Response:** Thank you for your suggestion, we have replaced the Figure 1 and with better quality of the graph.

**Conclusions**
**Reviewer #1:**

1. **This section needs to be revised with relevance to findings of the manuscript.L340-341-should it be part of results?**

**Response:** This section has been massively revised with relevance to the main findings. Some sentences apparently more suitable for Introduction or Result sections were removed from this section.
**2. L345-346- need justification for the statement for cross-reactivity.**

**Response:** After careful consideration, more accurate term to describe antigen’s reactivity to negative sera is specificity. So, we deleted the word of *cross-reactivity* from the manuscript.

**3.L357-359 should be part of introduction and not discussion.**

**Response:** We made great change to the Discussion section, and these sentences has been deleted from the manuscript.

**Reviewer #2:**

1. **The conclusions are supported the data presented and the authors have explained the public health relevance for human brucellosis by using the developed method (paper-based ELISA on multiepitope fusion protein)
   Response**: Thank you very much for your comments.

**Editorial and Data Presentation Modifications?**
Use this section for editorial suggestions as well as relatively minor modifications of existing data that would enhance clarity. If the only modifications needed are minor and/or editorial, you may wish to recommend “Minor Revision” or “Accept”.

**Reviewer #1:**

**Authors need to carefully review terminology used in the manuscript. The term “antigenicity” is misleading, when throughout the manuscript the fusion peptide was used as a target for detection of antibodies in human sera.**

**Response**: Thank you very much for your comment. In this paper, we discussed mainly the capability of fusion peptides in serodiagnosis of human brucellosis. To avoid misunderstanding, we deleted the word of *antigenicity* from manuscript, and replace it with other words such as diagnostic effect.
**Reviewer #2: Minor revision**

**Summary and General Comments**
**Reviewer #1: (No Response)**

**Reviewer #2:**

**1. This study is important to be published as the developed method will help the human brucellosis diagnosis.**

**Response:** Thank you very much for your comment.

**2. The study is novel and has shown the better performance in terms of sensitivity and specificity of the test.**

**Response:** Thank you very much for your comment.

**3. Please include the details of the ethical approval documents.**

**Response**: Before we applied for this project from National Natural Science Foundation of China in 2018, the application has been reviewed and approved by the Ethics Committee of Xuzhou Medical University. We attached the original ethical documents in picture format as following. At the same time, we also provided the translated version for your consideration.

**Translated version：**

**Medical Ethics Review Report**

School of Public Health of our school intends to carry out the scientific research work of *Study on the rapid diagnosis technology of human brucellosis based on multiepitope fusion protein*. This work involves the collection and detection of serum samples from human. The Ethics Committee of Xuzhou Medical University carefully reviewed the related medical ethics issues about this project.

**Project information:**

**Project name:** Study on rapid diagnosis technology for human brucellosis based on multiepitope fusion protein

**Institute undertaking this project:** Xuzhou Medical University

**Project leader:** Yin Dehui, Lecturer

**Start and end dates:** From January 2019 to December 2021

**Main content involved in human research:**

This project intends to use the serum of brucellosis patients together with bioinformatics, fusion protein, protein L and p-ELSIA technology to establish a rapid and sensitive new technology for the serological diagnosis of human brucellosis. It is of great significance for the prevention and treatment of the zoonotic infectious disease brucellosis in human, and also providing a valuable strategy for developing novel diagnostic technology for this disease.

**Review comments:**

After careful consideration by the Ethics Committee of Xuzhou Medical University, the research design and implementation fully comply with the principles of safety and fairness. The research content does not constitute any hazard factor to researchers and other populations, and there is no conflict of interest in the research content and results.

**Conclusion:**

In the study, the experimental content completely meets the requirements of medical ethics and there is no potential risk. Agreement is reached that this work can be carried out according to the plan.

Ethics Committee of Xuzhou Medical University

February 27, 2018

**Figure Files:**
While revising your submission, please upload your figure files to the Preflight Analysis and Conversion Engine (PACE) digital diagnostic tool, https://pacev2.apexcovantage.com. PACE helps ensure that figures meet PLOS requirements. To use PACE, you must first register as a user. Then, login and navigate to the UPLOAD tab, where you will find detailed instructions on how to use the tool. If you encounter any issues or have any questions when using PACE, please email us at figures@plos.org.

##### Response: We have uploaded all our figure files to the Preflight Analysis and Conversion Engine.   Data Requirements: Please note that, as a condition of publication, PLOS' data policy requires that you make available all data used to draw the conclusions outlined in your manuscript. Data must be deposited in an appropriate repository, included within the body of the manuscript, or uploaded as supporting information. This includes all numerical values that were used to generate graphs, histograms etc.. For an example see here: http://www.plosbiology.org/article/info%3Adoi%2F10.1371%2Fjournal.pbio.1001908#s5. Response: All data for our figure has been upload as supporting information.

##### Reproducibility: To enhance the reproducibility of your results, we recommend that you deposit your laboratory protocols in protocols.io, where a protocol can be assigned its own identifier (DOI) such that it can be cited independently in the future. Additionally, PLOS ONE offers an option to publish peer-reviewed clinical study protocols. Read more information on sharing protocols at https://plos.org/protocols?utm_medium=editorial-email&utm_source=authorletters&utm_campaign=protocols Response: Considering that this manuscript has been released as a preprint at Research Square (https://www.researchsquare.com/article/rs-291650/v1, DOI: 10.21203/rs.3.rs-291650/v1), we did not deposit it in protocols.io.

Once again, thank you very much for your comments and suggestions.

Best wishes,

##### Dehui Yin
